# Molecular mechanisms of detection and discrimination of dynamic signals

**DOI:** 10.1038/s41598-018-20842-y

**Published:** 2018-02-06

**Authors:** G. Antunes, A. C. Roque, F. M. Simoes-de-Souza

**Affiliations:** 1 Laboratory of Neural Systems (SisNe), Department of Physics, University of São Paulo, Ribeirão Preto, SP Brazil; 2 Center for Mathematics, Computation and Cognition, Federal University of ABC, São Bernardo do Campo, SP Brazil

## Abstract

Many molecules decode not only the concentration of cellular signals, but also their temporal dynamics. However, little is known about the mechanisms that underlie the detection and discrimination of dynamic signals. We used computational modelling of the interaction of a ligand with multiple targets to investigate how kinetic and thermodynamic parameters regulate their capabilities to respond to dynamic signals. Our results demonstrated that the detection and discrimination of temporal features of signal inputs occur for reactions proceeding outside mass-action equilibrium. For these reactions, thermodynamic parameters such as affinity do not predict their outcomes. Additionally, we showed that, at non-equilibrium, the association rate constants determine the amount of product formed in reversible reactions. In contrast, the dissociation rate constants regulate the time interval required for reversible reactions to achieve equilibrium and, consequently, control their ability to detect and discriminate dynamic features of cellular signals.

## Introduction

Cells detect endogenous signals through changes in the activities of biomolecules that integrate signalling pathways and networks^1–3^. Similarly, many drugs exert their effects by regulating components of signalling networks^4,5^. In recent decades, the advances of molecular biology and proteomics promoted a rapid growth in the understanding of the topological organization of signalling networks and pathways^2,6,7^. However, despite the wealth of data, the comprehension of the dynamics of interconnected biomolecules and how they underlie specific cellular processes in response to a vast variety of signals remain a challenge^2,5,6,8^.

Signalling pathways and networks typically show high numbers of cross-talks and redundancies^2,5,6,9^. Often, networks that share mutual components execute opposite cellular responses^10,11^. Moreover, common intracellular signals trigger several competing processes^9,10,12,13^.

To ensure the appropriate response to different signals, the activities of the biomolecules must be tailored to detect only the correct information^14^. Historically, the law of mass action extensively influenced our understanding of signalling transduction and the mechanisms of drug action^15,16^. In consequence, we tend to explain the activation of a molecule by a cellular signal or the effect of a drug as dose/concentration-dependent^15^. Thus, putative differences in the affinities for common activators is the typical explanation for the differential activations of competing signalling pathways^17,18^. Affinity is also a core concept in pharmacology, commonly used to predict the efficacy of drugs and lead compounds^15,16,19,20^. However, the concentrations of drugs and endogenous signals fluctuate constantly in the biological systems and often with faster time scales than the rates of binding and unbinding from their cellular targets^16,18,20^. Frequently the rate constants of the reactions play a more decisive role to their outcomes than thermodynamic parameters such as binding affinity^16,18,19,21^. Cumulating evidences have showed that the lifetime of a drug on its target is often more important for its physiological effects than the affinity of the drug/target complex^16,19^. Similarly, several biomolecules and signalling pathways detect the temporal dynamics of intracellular signals^13,14,18,22,23^, which implies that the concentrations of their activators are not the only property carrying information^2,13,14,22^. Therefore, one of the most important aspects of cellular signalling transduction that still needs to be addressed is the identification of the mechanisms that underlie the detection and discrimination of the dynamic features of cellular signals.

In this work, we used computational models that simulate the interactions between a ligand and different targets to characterize the role of kinetic and thermodynamic parameters in the detection and discrimination of dynamic signals. Our results indicated that only reactions outside mass-action equilibrium are sensitive to the temporal features of signal inputs. Consequently, their outcomes are not predicted by thermodynamic parameters such as binding affinities and dissociation constants. We also demonstrated that, outside mass-action equilibrium, the association rate constants regulate the amount of product formed in reversible reactions. The dissociation rate constants control the time required for reversible reactions to achieve equilibrium and determine their ability to detect and discriminate dynamic features of cellular signals. Moreover, in sequential reactions, fast dissociation rate constants act as bottlenecks for the propagation of dynamic signals.

## Results

### Mechanisms for the detection and discrimination of the durations of signals

Thermodynamic and kinetic parameters regulate chemical reactions, but their individual contributions vary^18,24^. For a reversible reaction of binding and unbinding (reaction 1) between a molecule M and a ligand L forming the complex LM:

chemical equation 1 $$L+M\rightleftarrows LM$$

the dissociation constant (K_D_) quantifies the binding affinity of the complex LM formed at equilibrium, which is mathematically defined by equation 1:

1 $${K}_{D}=\frac{[L][M]}{[LM]}$$

where the brackets indicate concentrations.

**Figure 1:**
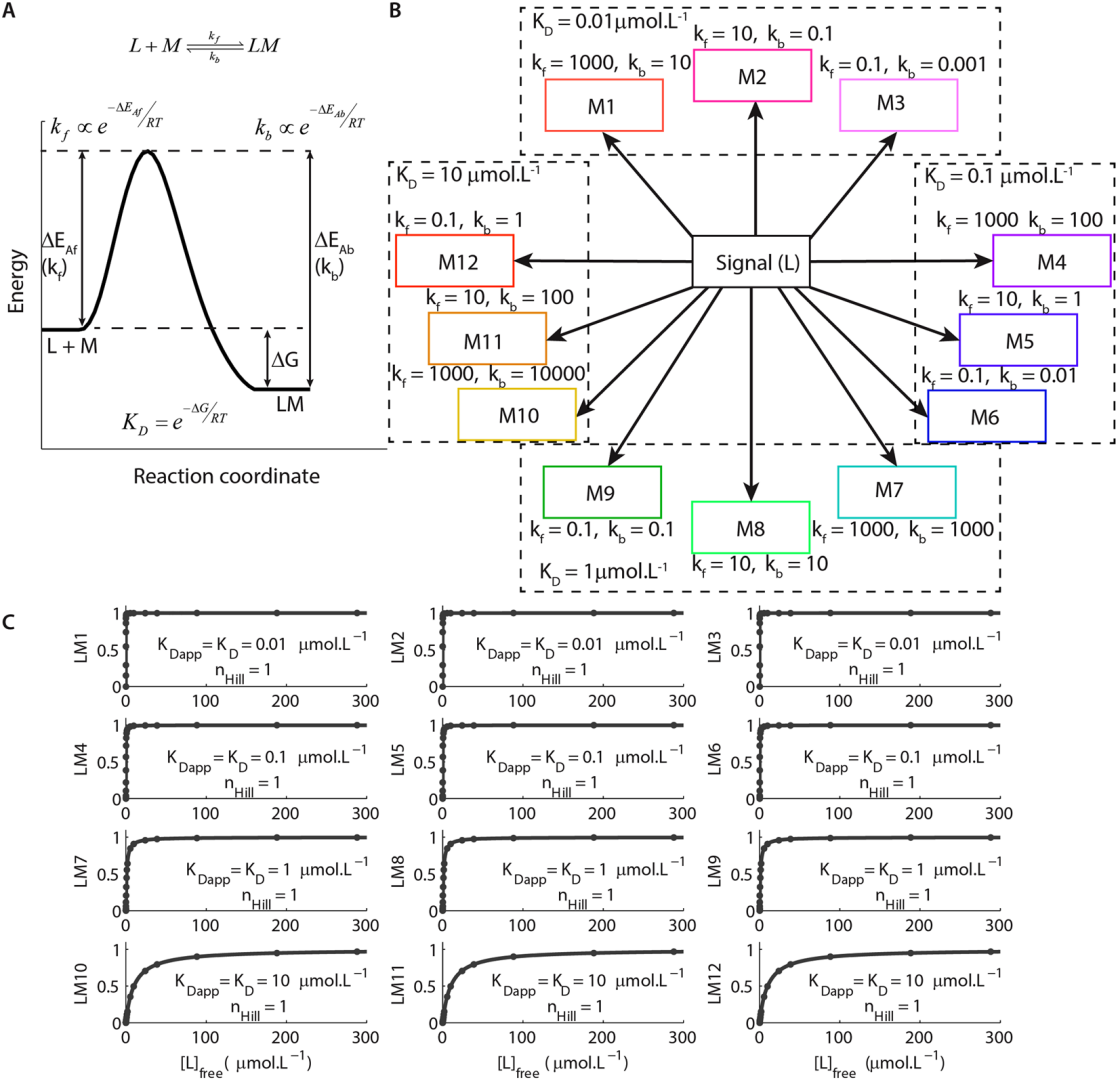
Thermodynamic and kinetic parameter of chemical reactions. (**A**) The energy profile for a simple reversible reaction of complex formation. The species L and M are the initial reactants, LM is the complex formed, k_f_ and k_b_ are the rate constants for the association (forward) and dissociation (backward) reactions, respectively. ΔE_Af_ and ΔE_Ab_ state for the energy of activation for the forward and backward reaction, respectively, *R* is the ideal gas constant, and *T* is the temperature in Kelvin. (**B**) Diagram of the simulated system, which consists of twelve different molecules (M1-12) interacting with a ligand (L) with different affinities (K_D_s) and rate constants (k_f_s are given in µmol.L^−1^.s^−1^ and k_b_s in s^−1^). (**C**) Dose-response curves for the formation of the complex LM1-LM12 as functions of [L]_free_. The K_Dapp_s estimated with these curves were set as the control K_D_s for the formation of LM1-LM12 in our simulations.

According to equation 1, the K_D_ of a reversible reaction specifies which species are more abundant at equilibrium (the reactants L and M or the product LM). The K_D_ of a reversible reaction is related with its Gibbs free energy (ΔG), which designates the stability of the product LM relative to the reactants L and M (Fig. 1A)^24,25^. As thermodynamic quantities, K_D_ and ΔG define the relative concentrations of its components at equilibrium, but do not indicate whether the reversible reaction occurs in a feasible time^24^. It is the energy barrier (energy of activation, E_A_) that must be overcome during a reaction that determines its velocity^24^. A low-energy barrier corresponds to a fast reaction and a high-energy barrier corresponds to a slow reaction (Fig. 1A). E_A_ regulates the rate constant (*k*) of a reaction, but not whether it is thermodynamically favourable^24^. When reactions occur at equilibrium, they are under thermodynamic control and regulated by thermodynamic parameters such as K_D_^24,25^. When they proceed outside equilibrium, they are under kinetic control and their rate constants determine their outcomes^24,25^.

In biological systems, the concentrations of drugs and endogenous signals vary often with time scales faster than the rates of binding and unbinding from their cellular targets^16,18,20^. In consequence, many cellular reactions do not achieve equilibrium or steady-state^16,18,20^. We hypothesised that only reactions that proceed outside mass-action equilibrium detect and discriminate dynamic cellular signals. To test this hypothesis, we simulated the interactions of twelve different molecules (M1-M12) with a ligand L to form the corresponding complexes LM1-LM12 (Fig. 1B). We simulated the formation of three complexes (LM1-LM3) with high affinity at equilibrium (K_D_ = 0.01 µmol.L^−1^), six with moderate affinity (LM4-LM6 with K_D_ = 0.1 µmol.L^−1^ and LM7-LM9 with K_D_ = 1 µmol.L^−1^) and three (LM10-LM12) with low affinity (K_D_ = 10 µmol.L^−1^). For each K_D_, we implemented three different sets of rate constants of association (k_f_) and dissociation (k_b_) to simulate reactions with varied velocities (Fig. 1B). We then obtained the dose-response curves for the formations of LM1-LM12 as functions of free concentrations of L ([L]_free_) at equilibrium (Fig. 1C) to ensure that the values of K_D_ used in the model matched the concentration of free ligand ([L]_free_) required to promote the half-maximum activation of each complex implemented, which we verified by fitting the equation (2):

2 $$A={A}_{{\rm{\max }}}\frac{{[L]}_{Free}^{{n}_{Hill}}}{{K}_{Dapp}^{{n}_{Hill}}+{[L]}_{Free}^{{n}_{Hill}}}$$

where A is the activity (i.e. normalized concentration) of the complexes LM1-LM12, A_max_ corresponds to their maximum activity (=1), the term n_hill_ is the Hill coefficient and K_Dapp_ is the apparent K_D_. As expected, independently of the rate constants used for the reactions simulated, the K_Dapp_s of the dose-responses corresponded exactly to the K_D_s implemented (Fig. 1C). We set these K_Dapp_s as the control K_D_s of LM1-LM12 hereafter. All curves presented n_Hill_ equal to 1.

Next, we used square pulses of [L]_free_ with different durations and peak concentrations to verify how the thermodynamic and kinetic parameters used regulate the detection and discrimination of dynamical signals, which we defined as the ability of molecules to respond and display different levels of activation to changes in the temporal properties of their signal activators. The durations and amplitudes of the pulses of [L]_free_ were set in the simulations in a non-conservative manner. Thus, the concentrations of L used in the pulses were buffered. Consequently, all molecules M1-M12 were exposed to the same signals and there was no competition among them.

**Figure 2:**
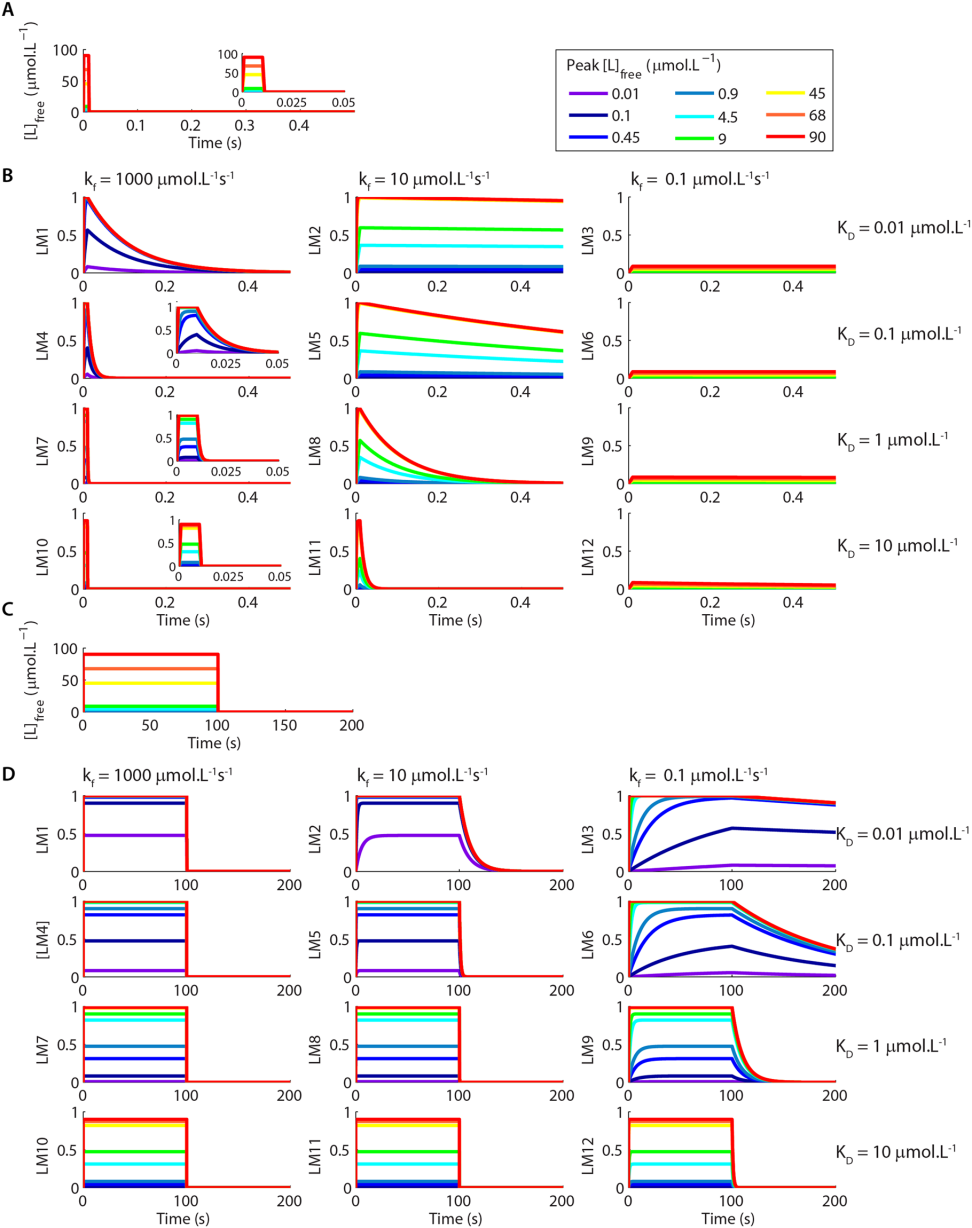
Examples of time courses of LM1-LM12 for pulses of [L]_free_ with different durations and concentrations. (**A,B**) Pulses of [L]_free_ of 10 ms of duration and varying concentrations (**A**) and the corresponding activations of LM1-LM12 (**B**). The insets show the results with different scales for better visualization. (**C,D**) Pulses of L with 100 s of duration and varying concentrations (**C**) and the activations of LM1-LM12 (**D**). The K_D_s for the formation of the complexes at equilibrium are showed on the right, and the association rate constants of complex formation (k_f_) on the top of the panels showed in B and D. The k_b_ for each reaction is calculated by: ${k}_{b}={k}_{f}\times {K}_{D}$. The legend indicates the colour code used to represent each concentration of [L]_free_.

The association and dissociation of complexes that have identical affinities at equilibrium proceeded with different time courses when we used square pulses of [L]_free_ as input signals (Fig. 2). Moreover, complexes that have the same K_D_ at equilibrium displayed different levels of activation (Fig. 2). These differences were strongly pronounced for short pulses, which possess durations within the range of pivotal cellular signals (varying from milliseconds to few seconds)^26–28^, and gradually disappeared as we stimulated the model with pulses that were long enough to allow the reactions to achieve equilibrium. For instance, LM10, LM11 and LM12 presented very different levels of activation when stimulated by brief pulses of [L]_free_ (10 ms) (Fig. 2A,B), but equivalent activations for pulses of [L]_free_ of 100 s (Fig. 2C,D).

To analyse these data, we used equation 2 to fit dose-responses curves of the peak concentrations of LM1-LM12 obtained as functions of the peak amplitudes of the pulses of [L]_free_ with different durations, and estimated the values of their K_Dapp_ and n_Hill_ for comparisons with the results of the system at equilibrium.

**Figure 3:**
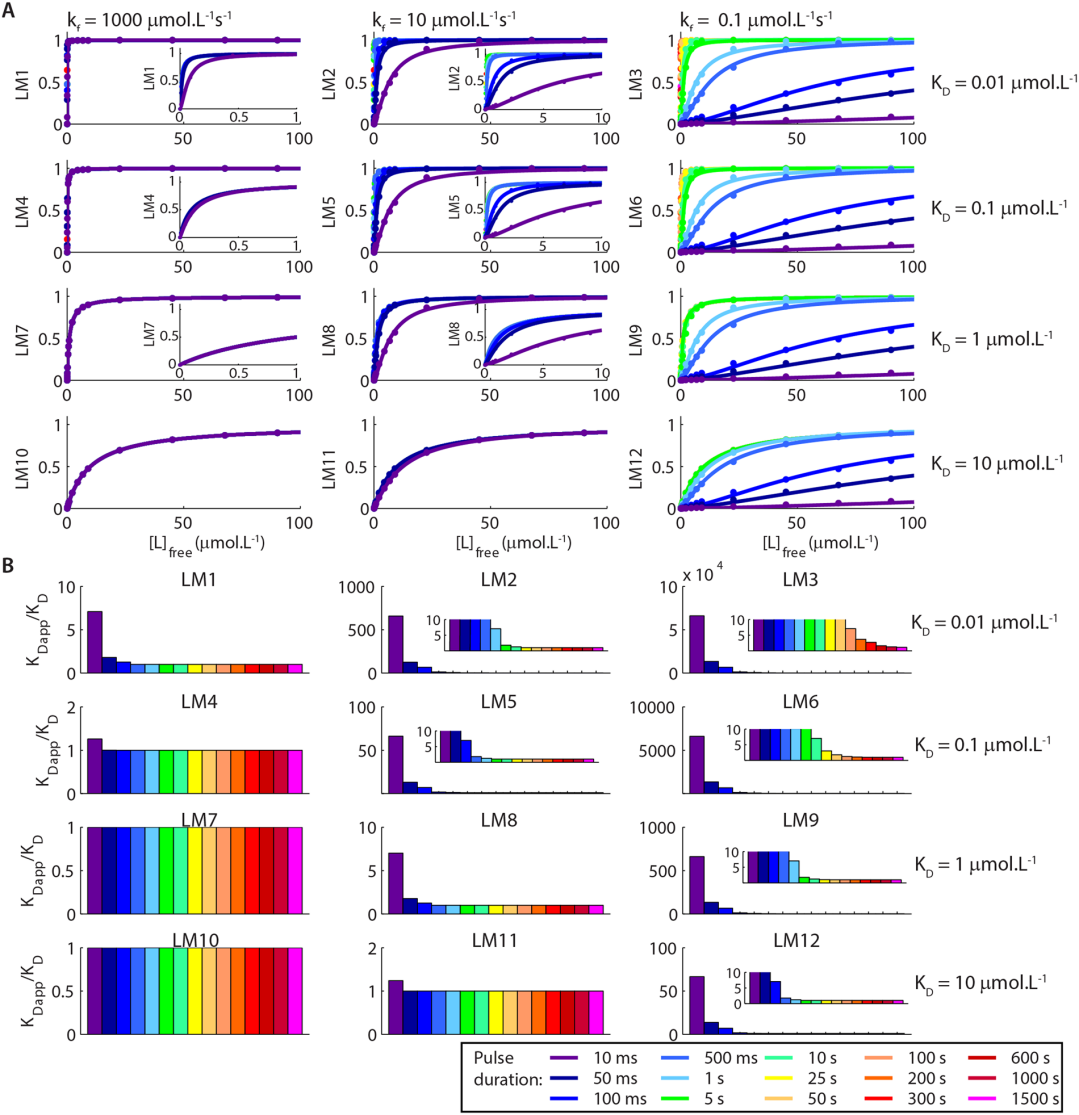
Activations of LM1-LM12 for pulses of [L]_free_ with different durations and concentrations. (**A**) Dose-response curves for the formations of LM1-LM12 as functions of pulses of [L]_free_ with different durations. (**B**) K_Dapp_/K_D_ ratios calculated using the K_D_s showed in Fig. 1C and the K_Dapp_s (Suppl. Table S1) obtained from the curves showed in **A**. The control K_D_s for the interaction of each molecule with L are showed on the right of the panels and the k_f_s for the association reactions are indicated on the top of **A**. The k_b_ for each reaction is calculated by: ${k}_{b}={k}_{f}\times {K}_{D}$. The insets show the same results in a different scale for better visualization. The legend indicates the colour code used to represent each duration of [L]_free_.

The dose-response curves of most complexes showed that the durations of pulses of [L]_free_ modulated their formations (Fig. 3A) by changing the values of K_Dapp_ in comparison to their control K_D_s in a duration-dependent manner. Figure 3B shows the K_Dapp_/K_D_ ratios to facilitate their comparisons, we listed the exact values of K_Dapp_ in Suppl. Table S1. As we increased the durations of pulses of [L]_free_, the values of K_Dapp_ decreased until they matched the control K_D_s (K_Dapp_/K_D_ = 1) indicating that the reversible reactions had reached equilibrium, which happened at different pulse durations for the molecules simulated (Fig. 3B). The dynamic changes of K_Dapp_s showed that the molecules detected the durations and the peak concentrations of the pulses of [L]_free_ by temporally integrating these signals over time. Once the durations of pulses of [L]_free_ were sufficiently long for the reactions to achieve mass-action equilibrium, they became insensitive to time and detected only the variations in the concentrations of L.

Our results demonstrated that the key point for the temporal discrimination of cellular signals relies on the different time scales in which each reversible reaction reach thermodynamic equilibrium. The longer it takes for a reaction to reach equilibrium, the larger is the range of durations of signals it can detect and discriminate by dynamically changing its K_Dapp_ (Fig. 3B).

The results of Fig. 3B also revealed that the dissociation rate constants (k_b_s) used in our simulations played a pivotal role in determining the time required for each reversible reaction to reach equilibrium (Fig. 3B). In a reversible reaction, the slower is the k_b_ the longer it takes for the activation of a given molecule to peak^29^. Our results indicated that, for the conditions that we simulated, the slower was the k_b_ the longer was the time interval required for the reactions to reach equilibrium independently of the k_f_ used. For instance, the reactions of formations of LM7 and LM10 occurred with very fast k_b_s in our simulations. Their formations proceeded at equilibrium for all pulse durations tested, consequently, they only detected the concentrations of L (Fig. 3B). However, the formation of LM1, which happened with the same k_f_ used for the formations of LM7 and LM10 but a slower k_b_, required pulses of 500 ms to exhibit K_Dapp_ compatible to its control K_D_. Reactions that have same k_b_s required identical durations of pulses of [L]_free_ to reach equilibrium independently of their k_f_s (Fig. 3B and Suppl. Table S1, compare the pairs LM1 and LM8, LM2 and LM9, LM5 and LM12). The complexes that dissociated with identical values of k_b_ also presented similar K_Dapp_/K_D_ ratios (Fig. 3B).

Outside the mass-action equilibrium, reversible reactions with identical values of k_f_ had equivalent numerical values of K_Dapp_ independently of their k_b_s and of their control K_D_s (Suppl. Table S1). For instance, the values of K_Dapp_ obtained for the formation of LM3 were much more similar to the K_Dapp_s of LM9 for most pulse durations tested than the K_Dapp_s of LM1 (Suppl. Table S1), even though LM1 and LM3 have identical K_D_s at equilibrium and the K_D_ of LM9 is 100-fold weaker. The larger were their k_f_s, the lower were their K_Dapp_s observed at non-equilibrium. This result indicates that complexes with faster k_f_s activate preferentially outside mass-action equilibrium. However, the closer the reversible reactions got to reaching equilibrium, the lesser their outcomes depended on their k_f_s and the more they depended on their thermodynamic affinities as expected^30^.

**Figure 4:**
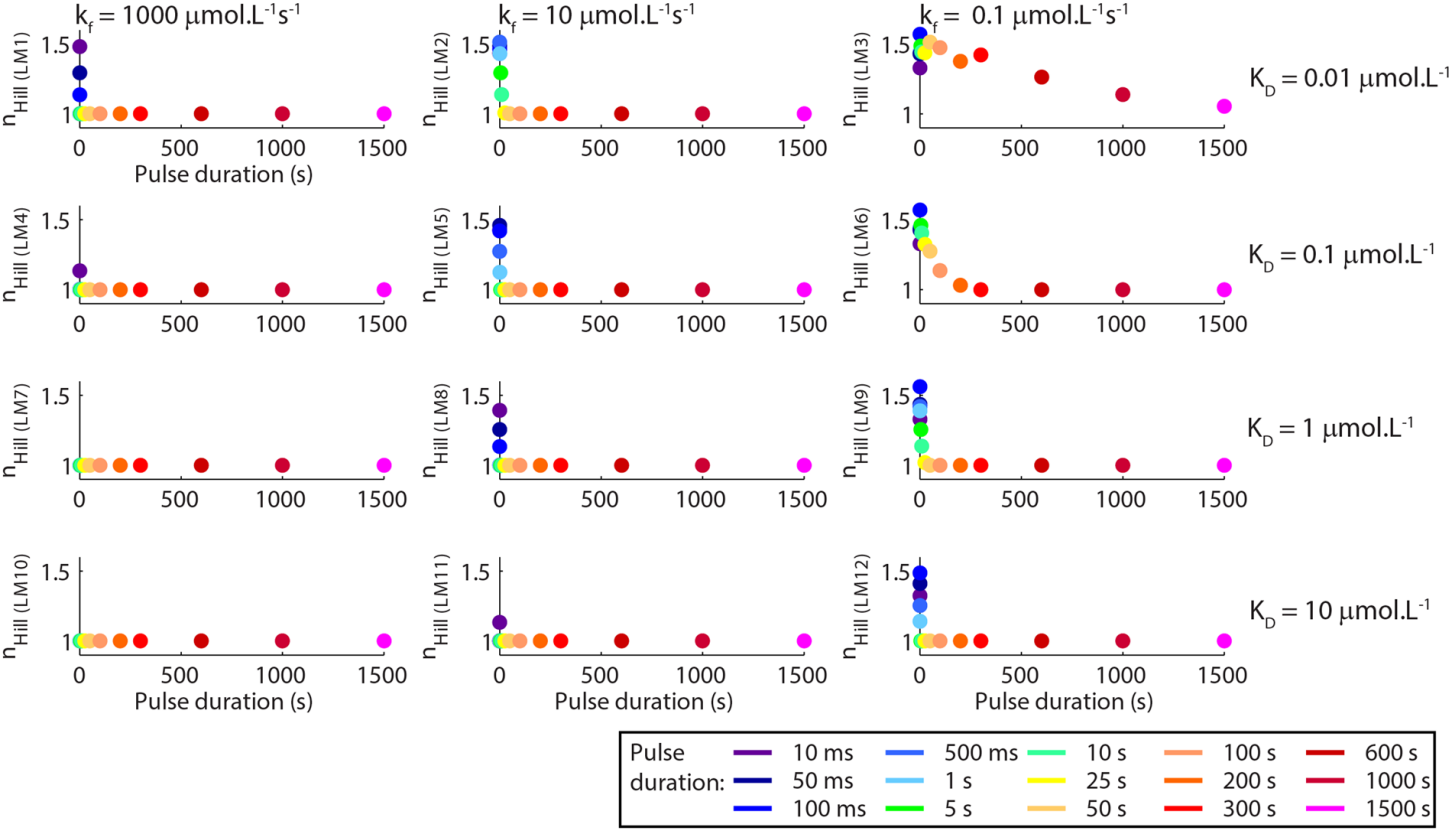
Values of n_Hill_ estimated from the dose-response curves showed in Fig. 3A.

In addition to the changes in K_Dapp_, we verified that durations of the pulses of [L]_free_ promoted variations in the n_Hill_ for the reactions that proceeded outside mass-action equilibrium, which displayed n_Hill_ larger than 1 even though the components of our system have no allosteric cooperativity (Fig. 4). The parameter n_Hill_ is commonly defined as an “interacting-coefficient” that reflects the cooperative binding of ligands to multiple sites of a molecule^31^. Nevertheless, it is important to note that, in addition to allosteric cooperativity, n_Hill_ larger than 1 can indicate ultrasensitivity. Multiple mechanisms promote ultrasensitivity including feedback loops, small changes in reactions near saturating conditions, distributive phosphorylations, among others^32–36^. In a ultrasensitivity system, n_Hill_ designates the degree of bistability^33,34,36^. In our results, we verified that the values of n_Hill_ became larger than 1 only for reactions happening outside mass-action equilibrium. For these reactions, the values of n_Hill_ increased as we reduced the durations of pulses of [L]_free_. The changes of n_Hill_ resulted from ultrasensitivity promoted by the filtering of fast signals with low amplitudes as if they were noise. Previously, it was proposed that biology evolved to use non-equilibrium to efficiently discriminate signals from noise^16^, which is consistent with our results. We had observed similar changes of n_Hill_ previously^18^.

### Detection and discrimination of frequencies and number of pulses of dynamic signals

**Figure 5:**
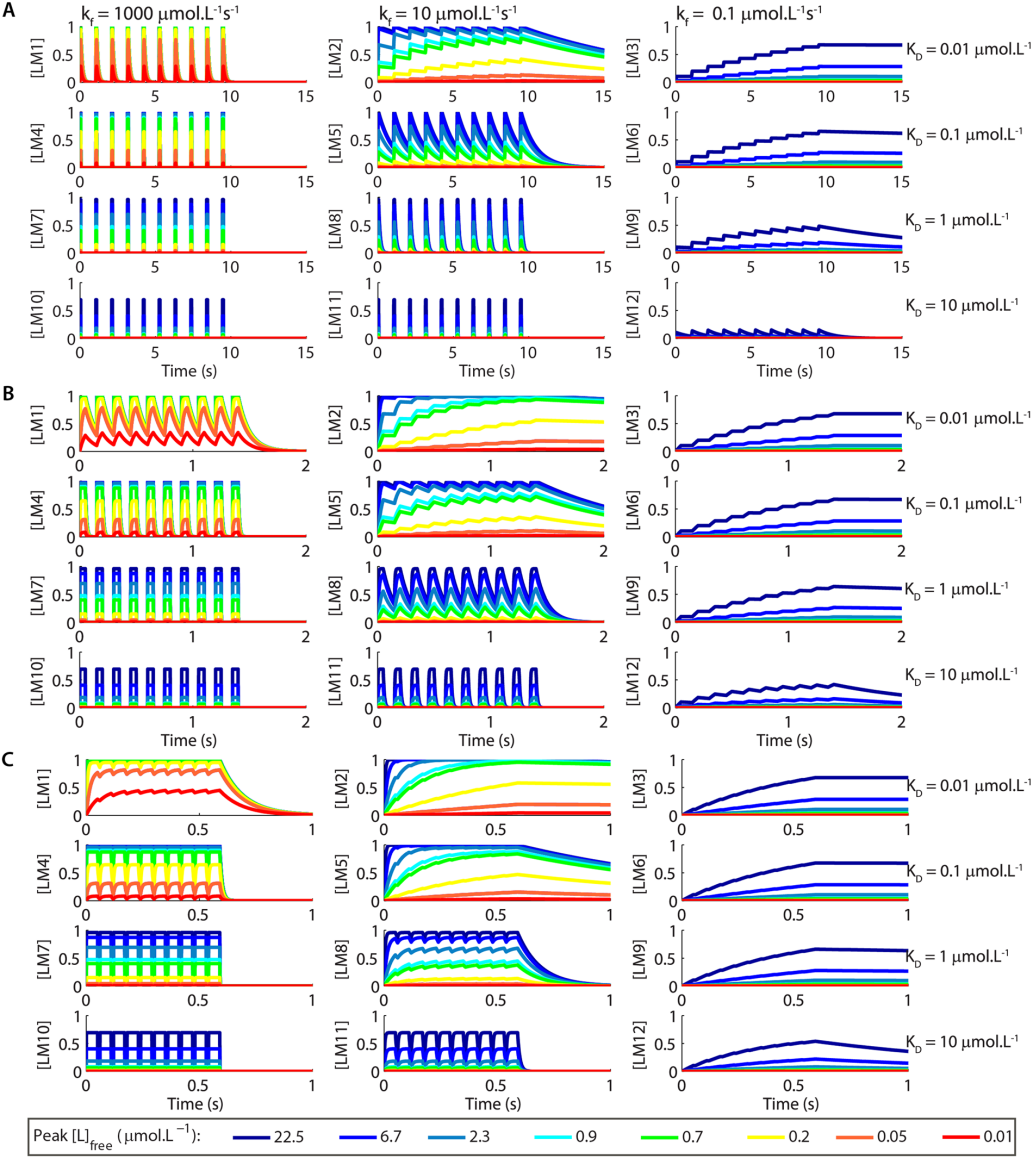
Examples of the time courses for the formation of LM1-LM12 obtained using trains of ten pulses of [L]_free_ with different peak amplitudes released at 1 Hz (**A**), 10 Hz (**B**), or 100 Hz (**C**). Each pulse of [L]_free_ had 50 ms of duration. The time courses of [L]_free_ are showed in Suppl. Fig. S1A–C. The control K_D_s for the interactions of M1-M12 with L are showed on the right of the panels and the k_f_s for the association reactions are indicated in **A**. The legend indicates the colour code used to represent each concentration of [L]_free_.

Next, we investigated how the kinetic and thermodynamic parameters underlie the discrimination of interpulse intervals and number of pulses of trains of signals of L, a property displayed by several enzymes and signalling pathways^18,22,23,37^. We stimulated the formation of LM1-LM12 with trains of ten pulses of [L]_free_ delivered at 1 Hz (1 s of interpulse interval), 10 Hz (100 ms of interpulse interval), or 100 Hz (10 ms of interpulse interval). Each pulse had duration of 50 ms (Suppl. Fig. S1A–C) or 100 ms (Suppl. Fig. S1D–F). Figure 5 and Suppl. Fig. S2 show examples of the time courses of LM1-LM12 observed. To verify whether the formation of LM1-LM12 detected the interpulse interval and the number of pulses of L simulated, we measured the peak amplitude of LM1-LM12 formed as functions of the peak of each pulse of [L]_free_ within a train (Suppl. Fig. S3). We used these data to fit ten dose-response curves for each frequency tested using equation 2 (Suppl. Figs S4 and S5). Each curve corresponded to the formations of LM1-LM12 observed for a specific pulse number (Suppl. Figs S4 and S5). With these curves, we investigated whether the values of K_Dapp_ and n_Hill_ varied during each train and quantified their discrepancies from the control K_D_s (Fig. 1C).

**Figure 6:**
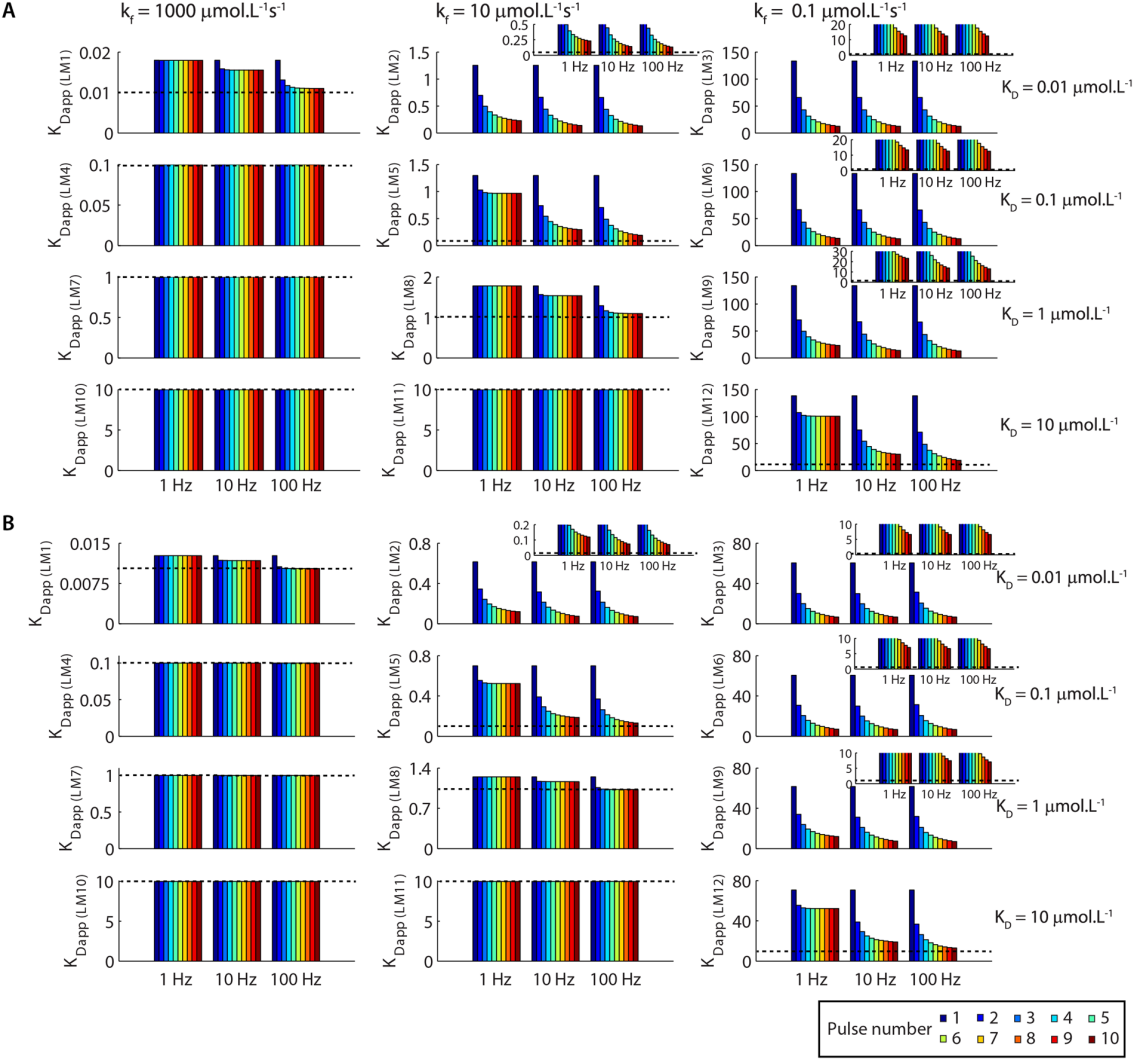
Variations of the K_Dapp_s observed for the stimulations of the model with trains of pulses of [L]_free_. We estimated the K_Dapp_s (in µmol.L^−1^) from dose-responses curves showed in Suppl. Figs S4 and S5. The results showed in (**A)** and (**B)** were obtained for pulses of 50 ms and 100 ms, respectively. The dashed lines correspond to the control K_D_s, also indicated on the right of the panels, the k_f_s for the association reactions are indicated in **A**. The insets show the same results in different scales for better visualization. The legend shows the colour code used to represent the pulse number of [L]_free_.

The K_Dapp_s obtained (Fig. 6A and B) revealed different types of dynamic signal discriminations that relied heavily on the k_b_s used in the reactions of the different complexes. The K_Dapp_s for the formations of LM4 (k_b_ = 100 s^−1^), LM7 (k_b_ = 1000 s^−1^), LM10 (k_b_ = 10000 s^−1^) and LM11 (k_b_ = 100 s^−1^) corresponded to the control K_D_s to all situations tested and demonstrated that these complexes were insensitive to the number of pulses, the interpulse intervals and the durations of pulses used in the simulations. However, as we decreased the k_b_s, this scenario changed.

The K_Dapp_s of LM1 and LM8, which had k_b_ = 10 s^−1^, detected and discriminated mainly the interpulse interval between the signals of [L]_free_ used. Moreover, we observed changes of their K_Dapp_s during the initial pulses of [L]_free_ released at 10 Hz and 100 Hz. Thus, LM1 and LM8 also discriminated a limited number of pulses released at intermediary or high frequencies, because their interpulse intervals were shorter than the time required for the inactivation of both complexes. Consequently, there were summations of their activations for the initial pulses of trains released at 10 Hz and 100 Hz (Figs 5 and S2), which promoted alterations in their K_Dapp_s (Fig. 6A and B). In contrast, pulses released at 1 Hz had a long interpulse interval (1 s) that prevented the accumulation of LM1 and LM8 from one pulse to another (Figs 5 and S2). The K_Dapp_s of LM1 and LM8 matched the control K_D_s when stimulated with 3 or more pulses of L with 100 ms of duration released at 100 Hz.

LM5 and LM12 (k_b_ = 1 s^−1^) exhibited K_Dapp_s that changed as functions of both the interpulse interval and the number of pulses of [L]_free_ released at 10 Hz and 100 Hz. For signals of [L]_free_ released at 1 Hz, the K_Dapp_s of LM5 and LM12 changed only during the initial four pulses (Fig. 6A and B). Thus, LM5 and LM12 acted as good detectors and discriminators of the interpulse intervals for all frequencies tested and of the number of pulses of [L]_free_ released at moderate to high frequencies, but poor detectors of the number of pulses released at a low frequency (Fig. 6A and B).

The K_Dapp_s of LM2 and LM9 (k_b_ = 0.1 s^−1^) detected and discriminated the number of pulses of [L]_free_ for all frequencies tested. In addition, LM2 and LM9 discriminated the interpulse interval of pulses released at 1 Hz from pulses released at 10 Hz or 100 Hz. However, their K_Dapp_s did not discriminate the interpulse interval of pulses released at 10 Hz from pulses released at 100 Hz (Fig. 6A and B).

LM3 and LM6, the two complexes with the slowest k_b_s implemented (k_b_ = 0.001 s^−1^ and 0.01 s^−1^, respectively), presented K_Dapp_s that discriminated the number of pulses of [L]_free_, but were insensitive to their interpulse intervals (Fig. 6A and B).

None of the K_Dapp_s of LM2, LM3, LM5, LM6, LM9 and LM12 matched their control K_D_s (Fig. 6A and B, black dashed lines) evidencing that they did not reach mass-action equilibrium in the situations simulated. Moreover, the values of n_Hill_ for all the complexes that did not present values of K_Dapp_ compatible with the control K_D_s were larger than 1, which indicated that their activations had a bistability not observed at equilibrium (Suppl. Fig. S6). The k_b_s used in the simulations regulated the K_Dapp_/K_D_ ratio, as observed in the previous session. Hence, molecules with identical k_b_s (LM1/LM8, LM2/LM9, LM5/LM12) exhibit K_Dapp_s that diverged from K_D_ with equivalent magnitudes (Suppl. Fig. S7).

### Detection and discrimination of dynamic signals in sequential reactions

Next, we explored the detection and discrimination of dynamic signals in sequential reactions. Firstly, we implemented the reactions of association/dissociation of LM1-LM12 with the targets T_1_-T_12_ using one set of rate constants (k_f_ = 10 µmol^−1^.L.s^−1^, k_b_ = 0.1 s^−1^, K_D_ = 0.01 µmol.L^−1^). Specifically, LM1 reacted with T_f1,_ LM2 with T_f2,_ LM3 with T_f3,_ and so on, which resulted in twelve ternary complexes LM1T_1_-LM12T_12_ formed according to the sequential reactions:

chemical equation 2 $$L+Mn\underset{{k}_{b}}{\overset{{k}_{f}}{\rightleftharpoons }}LMn+{T}_{n}\underset{0.1{s}^{-1}}{\overset{10\,\mu mol.{L}^{-1}.{s}^{-1}}{\rightleftharpoons }}LMn{T}_{n}$$

where n = 1, 2, 3, …, 12. The parameters k_f_ and k_b_ refer to the rate constants used for the association/dissociation of LM1-LM12 (Fig. 1B).

In a different set of simulations, we implemented the interactions of LM1-LM12 with the targets T′_1_-T′_12_ to form LM1T′_1_-LM12T′_12_ using a different set of rate constants (k_f_ = 0.1 µmol^−1^.L.s^−1^, k_b_ = 0.001 s^−1^, K_D_ = 0.01 µmol.L^−1^), but equivalent sequential reactions:

chemical equation 3 $$L+Mn\underset{{k}_{b}}{\overset{{k}_{f}}{\rightleftharpoons }}LMn+T{\text{'}}_{n}\underset{0.001{s}^{-1}}{\overset{0.1\,\mu mol.{L}^{-1}.{s}^{-1}}{\rightleftharpoons }}LMn{T^{\prime} }_{n}$$

**Figure 7:**
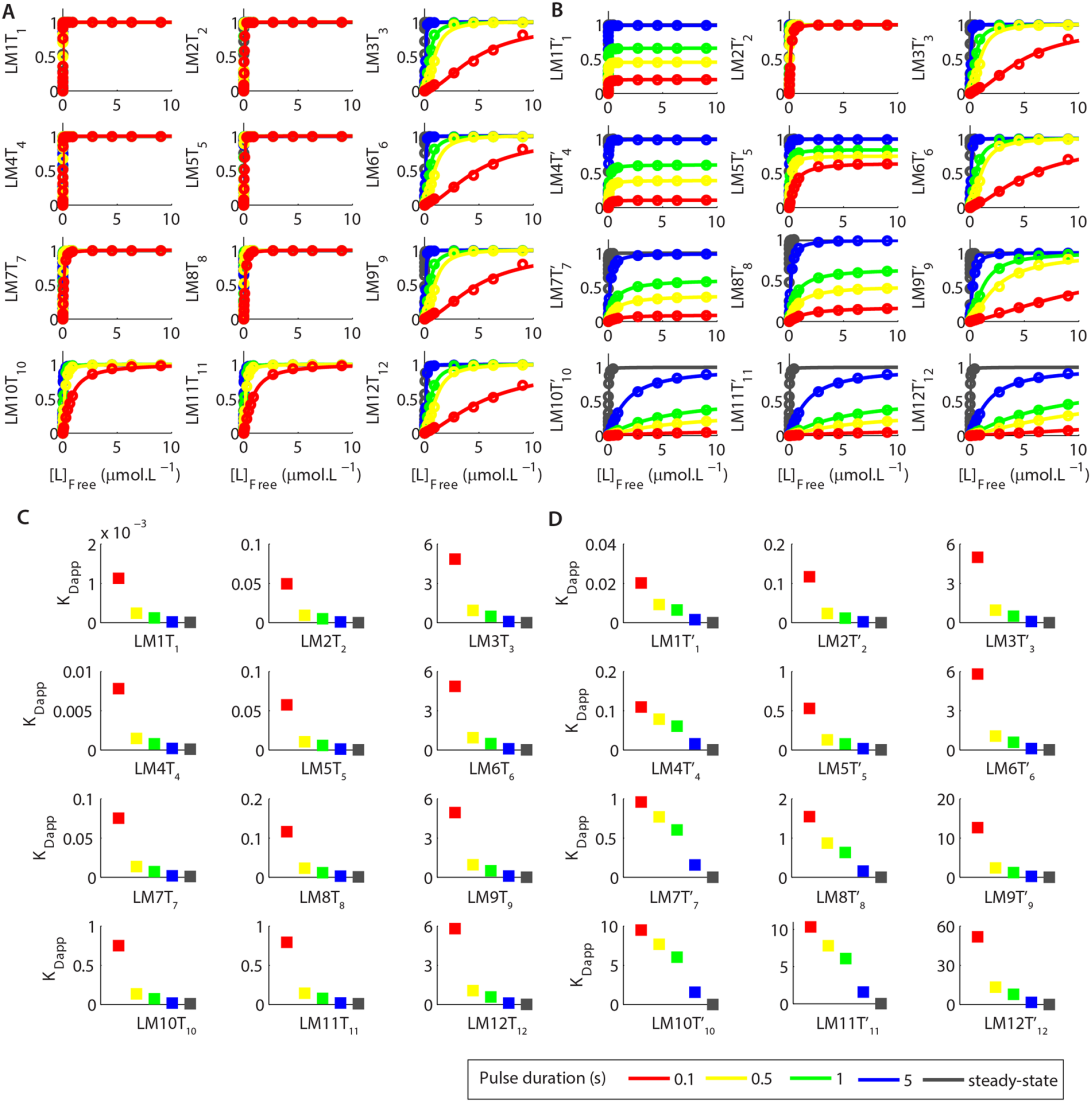
Detection and discrimination of dynamic signals by sequential reactions. (**A**) Dose-response curves of the complexes LM1T_f1_-LM12T_f12_ as functions of [L]_free_ with different durations. (**B**) Dose-response curves of the complexes LM1T′_1_-LM12T′_12_ as functions of [L]_free_ with different durations. (**A**,**B**) Values of K_Dapp_ estimated for the activation of LM1T_f1_-LM12T_f12_ (**C**) and LM1T′_1_-LM12T′_12_ (**D**) for pulses of [L]_free_ with different durations. The legend indicates the colour code used to represent durations of the pulses of [L]_free_.

Initially, we simulated the formations of LM1T_1_-LM12T_12_ and LM1T′_1_-LM12T′_12_ at steady-state as functions of different [L]_free_ to obtain dose-response curves fitted with equation 2 and estimate the control K_D_s and n_hill_. Note that the K_D_ used for the interactions of LM1-LM12 with T_1_-T_12_ and with T′_1_-T′_12_ are identical (K_D_ = 0.01 µmol.L^−1^). Nevertheless, the control K_D_s for the formations of LM1T_1_-LM12T_12_ and LM1T′_1_-LM12T′_12_ as functions of [L]_free_ varied according to the K_D_ of their binary precursors. Thus, the ternary complexes formed by LM1, LM2 and LM3 (LM1T_1_, LM2T_2_, LM3T_3_, LM1T′_1_, LM2T′_2_ and LM3T′_3_) exhibited control K_D_s as functions of [L]_free_ of approximately 0.00001 µmol.L^−1^ (Fig. 7, Suppl. Table S2), which is 1000-fold lower than the control K_D_s of their binary precursors (Suppl. Table S1). The ternary complexes formed by LM4, LM5 and LM6 (LM4T_4_, LM5T_5_, LM6T_6_, LM4T′_4_, LM5T′_5_ and LM6T′_6_) had control K_D_s as a function of [L]_free_ of 0.0001 µmol.L^−1^, which is higher than the K_D_s of the ternary complexes LM1T_1_-LM3T_3_ and LM1T′_1_-LM3T′_3_, but is also 1000-lower than the K_D_s for the formations of their precursors LM4, LM5 and LM6 (Fig. 7, Suppl. Table S2). The same pattern was also observed for the other ternary complexes simulated. Consequently, all the ternary complexes exhibited control K_D_s for their activations as functions of [L]_free_ at steady-state approximately 1000-fold lower than the K_D_s of their binary precursors, which demonstrated that the binding of each binary complex to a target affected its interaction with L. This type of alteration is commonly observed in biological systems^18,38^.

Next, we used square pulses of [L]_free_ with different durations (100 ms, 500 ms, 1 s, and 5 s) and peak concentrations to investigate how they regulated the formations of the ternary complexes LM1T_1_-LM12T_12_ and LM1T′_1_-LM12T′_12_. The results obtained were used to trace dose-responses curves of the peak concentrations of LM1T_1_-LM12T_12_ and LM1T′_1_-LM12T′_12_ as functions of the peak [L]_free_ using equation 2. The curves were used to verify whether the ternary complexes simulated detected and discriminated the durations of the signals of [L]_free_ by changing their values of K_Dapp_, n_Hill_ and maximum activation (A_max_) in comparisons to the values observed at steady-state (Fig. 7A,B).

In the previous sessions, we demonstrated that the rate constants used for the interactions of L with its targets M1-M12 modulated their K_Dapp_ and n_Hill_. The results showed in Fig. 7A,B revealed that the rate constants used in the reactions of M1-M12 with L can also modulate the values of A_max_ obtained for the dose-response curves of the formations of their respective ternary complexes. Thus, binary precursors that dissociated with fast k_b_s (≥1 s^−1^) from L impaired the A_max_ observed for the activation of their corresponding ternary complexes. However, such impairment only occurred for the ternary complexes formed with slow k_f_ (0.1 µmol^−1^.L.s^−1^), which indicates that it is the combination of the k_b_ of the precursor with the k_f_ for its interaction with its target that regulates A_max_ (Fig. 7B) and, in addition, also affected the values of K_Dapp_ and n_Hill_ (Fig. 7C,D and Suppl. Fig. S8).

Our results demonstrated that all ternary complexes simulated decoded the pulses duration tested and exhibited changes in their K_Dapp_ values in comparisons to their control K_D_ values observed at steady-state (Fig. 7C,D). Yet, all the ternary complexes that exhibited impairments of A_max_ for the durations of pulses of [L]_free_ also showed higher shifts in their values of K_Dapp_ in comparison to the values observed at steady-state (Fig. 7C,D). Thus, the combination of short half-lives of fast dissociating binary precursors greatly impaired the formation of ternary complexes that associate with slow k_f_. For instance, the binary complexes LM4, LM5 and LM6, which share the same control K_D_, dissociated with k_b_ of 100 s^−1^, 1 s^−1^ and 0.01 s^−1^, respectively. Due to the fast inactivation rate of LM4, the dose-response curves of activation of LM4T′_4_ as functions of pulses of [L]_free_ with different durations exhibited strong modulations of A_max_, but a similar modulation was not observed for the activation of LM4T_4_, which reacted faster with its precursor. Moreover, LM4T′_4_ exhibited much higher values of K_Dapp_ than LM4T_4_ for the pulses of [L]_free_ tested though both species have the same binary precursor and identical control K_D_. The curves for the activations of LM5T′_5_ showed a similar pattern observed for the curves of LM4T′_4_, but with less pronounced modulations as its binary precursor had a slower k_b_. LM5T′_5_ also exhibited higher values of K_Dapp_ than LM5T_5_, though both complexes have identical control K_D_ values and interact with the same binary precursor. In contrast, the dose-response curves of LM6T′_6_ had no variation in their A_max_ because its precursor, LM6, had a slow k_b_. In addition, the values of K_Dapp_ verified for LM6T_6_ were very similar to the values observed for LM6T′_6_. Thus, the slow time course for the inactivation of LM6 allow it to act as a “molecular memory” and propagate the transient signals of L for longer periods in comparison to LM4 and LM5.

In Suppl. Fig. S8A and B we plotted the n_Hill_ obtained for each dose-response curve showed in Fig. 7A and B. Our results demonstrated that, outside mass-action equilibrium, the formations of LM1T_1_-LM12T_12_ showed higher values of n_Hill_ in comparison to their binary precursors LM1-LM12, which indicated an increase in bistability along the sequential reactions simulated caused exclusively by kinetic factors (Suppl. Fig. S8A). Nevertheless, for the ternary complexes LM1T′_1_-LM12T′_12_, the curves that presented impairments of A_max_ exhibited values of n_Hill_ close to 1 and often lower than the values observed for their binary precursors (Suppl. Fig. S8B). These results indicated that, when we used pulses of [L]_free_ with different durations to promote the formation of LM1T′_1_-LM12T′_12_, the short half-life of binary precursors with fast k_b_s impaired their activations and affected not only their A_max_ and K_Dapp_, but also their values of n_Hill_.

### Detection and discrimination of dynamic signals in competing systems

**Figure 8:**
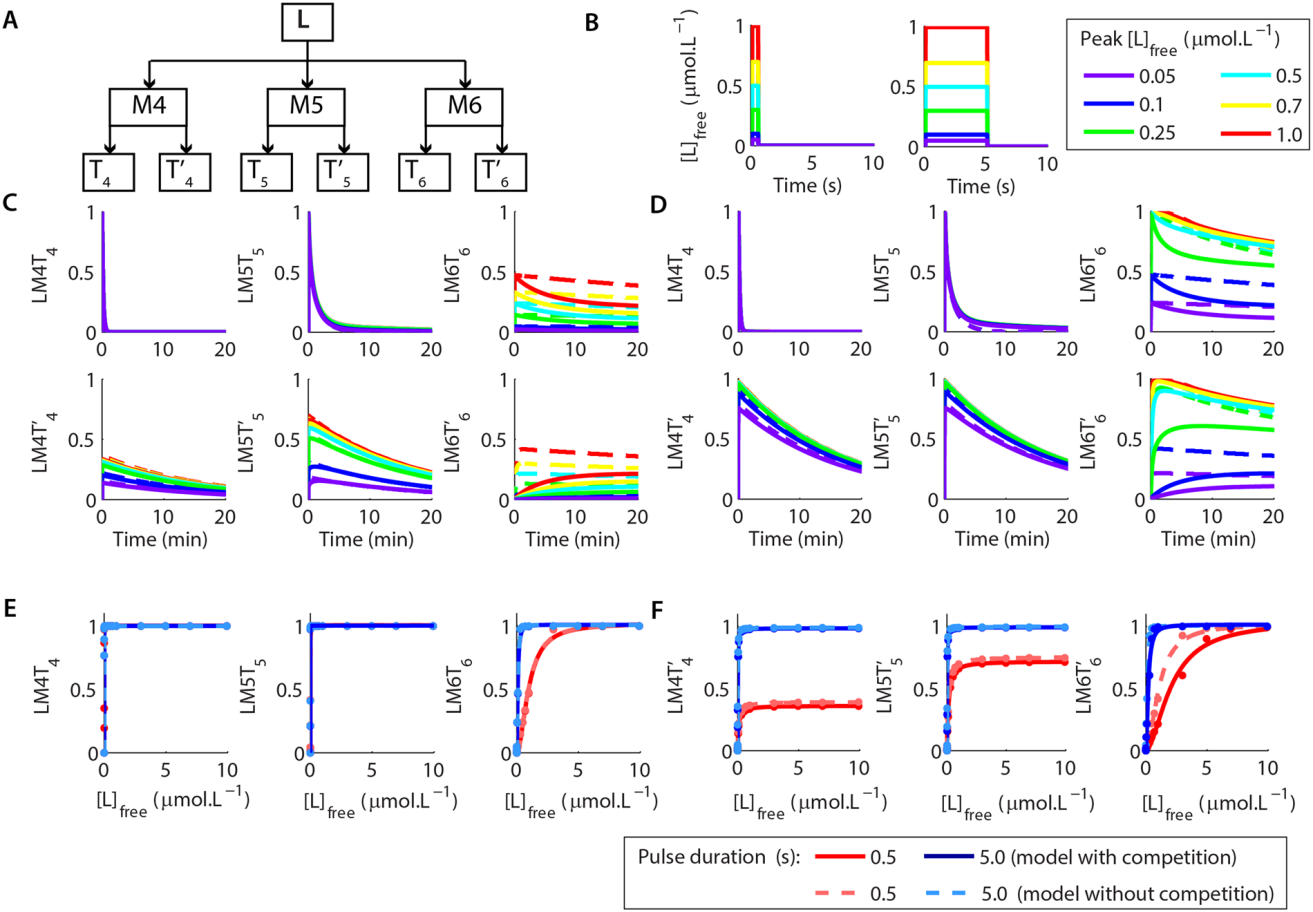
The role of competition in the detection and discrimination of dynamic signals. (**A**) The simulated system consisted of 3 targets activated by L (M4, M5, and M6) forming the complex LM4, LM5 and LM6. Each complex activated two substrates in a competitive manner. (**B**) We used pulses of [L]_free_ with different durations (0.5 and 5 s) and peak concentrations to verify the role of competition in the activation of the targets of LM4, LM5, and LM6. (**C,D**) The results obtained showed that competition did not affected the peak activation of fast-reacting targets (LM4T_4_, LM5T_5_, LM6T_6_) for pulses of [L]_free_ with 0.5 s (**C**) or 5 s (**D**) of duration in comparison to the results without competition (dashed lines). However, the presence of competition impaired the peak activation of slow reacting-targets (LM4T′_4_, LM5T′_5_, LM6T′_6_), promoting a slightly reduction in the peak amplitude of their dose-response curves of activation and a shift in their values of K_Dapp_ (**E-F**), which became larger (Suppl. Table S3).

The last stage of our work consisted in investigating how competition among different molecules shapes their response. For this analysis, we used a simplified version of our system containing only the formation of LM4, LM5 and LM6, which exhibit very distinct patterns of activation even though they share the same control K_D_. Each one of this species were responsible for the activation of two targets simulated as described in the previous session. However, for this stage of the work, we simulated the two targets activated by each LM complex competing for their activators (Fig. 8A). We then used pulses of L with different durations (0.5 s and 5 s) to verify the consequences of competition in the results previously described (Fig. 8B–D).

The competition for a common activator had two consequences in our system. For the formation of fast-reacting ternary complexes (LM4T_4_, LM5T_5_, and LM6T_6_), independently of the pulse duration tested, the competition with slow-reacting ternary complexes (LM4T′_4_, LM5T′_5_, and LM6T′_6_) did not affect the maximum amplitude of activation for each pulse tested, but accelerated the inactivation of LM6T_6_ (Fig. 8B–D). In contrast, for the slow-binding complexes (LM4T′_4_, LM5T′_5_, and LM6T′_6_), the presence of competition affected their maximum activation, which reduced slightly the A_max_ of their dose-response curves of activation (8E-F, Suppl. Table S3). Moreover, for LM6T′_6_ competition promoted a delay in its activation curves (Fig. 8B–D) and shifted the K_Dapp_ of its dose-response curves (8E-F). Taken together, these results indicated that the effects of competition vary depending on the combination of the half-life of the initial precursor (LM4, LM5, and LM6) with the rates of activation of the subsequent molecules.

## Discussion

Time is an important variable in the biological environments. The temporal dynamics of cellular signals regulate many molecules and signalling networks^13,14,18,22,23,37^. However, the comprehension of the molecular mechanisms that underlie the temporal regulation of cellular processes remains a challenge because much of our understanding of signalling processes results from data obtained at equilibrium or steady-state conditions. In this work, we focused on the identification of the molecular mechanisms that underlie the detection and discrimination of the temporal features of signals. For that, we simulated reactions of association and dissociation between molecules and a ligand. Previously, we used realistic models of different biomolecules with intricate interactions with endogenous ligands to investigate their modes of activation^18^. However, the level of complexity of our previous work made the comparison among different molecules difficult. Thus, in this work we have opted to use a generic and simpler system.

Biological systems are open systems in constant change^16,18,19^. The concentrations and levels of activation of biomolecules fluctuate continually, which sets perfect conditions for several reactions to proceed at non-equilibrium^16,18,19^. Consequently, many reactions in biological systems detect and discriminate dynamic signals and use temporal properties to display differential patterns of activation^13,14,22,23,37^. Because these reactions are sensitive to the temporal features of their components, they are under kinetic control and thermodynamic parameters such as K_D_ do not predict their outcomes, which is a conclusion fully supported by our results.

Several data have revealed that the association and dissociation rate constants (k_f_ and k_b_, respectively) for the interaction between biomolecules or of a drug with its targets are often more important than the binding affinity of the resulting complexes^19,21,39,40^. However, though in some systems the values of k_f_ are crucial^21^, especially for the interaction of drugs with their targets the k_b_ appears to play a more fundamental role^19,40^. Our results indicated that both rate constants are important in the detection of dynamical signals because they play different roles. At non-equilibrium, the k_f_s used in our simulations played a predominate role in determining the levels of activation of the ligand/molecule complexes simulated. The faster were k_f_s, the lower were the K_Dapp_s obtained, which indicate that molecules with fast k_f_s activate better at non-equilibrium. Similar results were observed previously^21^. Our results showed that the affinities observed at equilibrium do not ensure which molecules will activate in larger amounts^18,19,21^. A high affinity complex with slow rate constants can display a K_Dapp_ equivalent to the K_Dapp_ of a weak affinity complex when their reactions occur outside mass-action equilibrium. Only when the reactions approach mass-action equilibrium their rate constants become less important and their outcomes gradually become defined by their control K_D_s^30^.

In our simulations, the k_b_s played a pivotal role determining the time required for each reaction to achieve equilibrium. The slower was the k_b_ used, the larger was the range of signal durations that a reaction detected and discriminated and the longer was the time required for it to reach equilibrium. Slow k_b_s also promoted reactions sensitive to the frequencies and number of pulses of reacting signals. Nevertheless, the slower was the k_b_ used in our simulations, the better the reactions detected the number of pulses despite of their interpulse interval, which indicates that reactions with slow k_b_s integrate the signals over time more efficiently. These results contribute to explain why some molecules are sensitive to the interpulse interval of their signals, while others count pulses of signals regardless of their frequencies^18,23^. In addition, this type of information is crucial for the design of artificial signalling systems and probes^41,42^. We also demonstrated that the k_b_s played a crucial role in the propagation of dynamic signals. Thus, ligand/molecule complexes that dissociate with fast k_b_s do not propagate efficiently fast signals for slow-interacting targets. Consequently, in this scenario, complexes that dissociate slowly propagate dynamic signals better. Several observations have demonstrated that often drugs that dissociate slowly from their endogenous targets are more efficient, though the reasons for this process are not totally understood^19,40^. In this work, we have not explored this process specifically. The time intervals of the dynamic signals that we investigated are more compatible with physiological signals. However, our observations are not restricted to endogenous molecules and might explain the role of rate constants on the efficacy of drugs as well.

## Methods

We implemented the computational models using BioNetGen^43^, a rule-based software for modelling signaling networks and pathways. All simulations were solved deterministically.

To define the parameters of the model, we used K_D_s (0.01 µmol.L^−1^, 0.1 µmol.L^−1^, 1 µmol.L^−1^ and 10 µmol.L^−1^) commonly found for the interactions between biomolecules^10,18,38,44,45^. We defined the kinetic parameters of the model by setting a k_f_ of 1000 µmol^−1^.L.s^−1^ as our upper limit, which is consistent with the second order rate constant of a diffusion limited reaction in the cellular milieu. The other two k_f_s used in the model (10 µmol^−1^.L.s^−1^ and 0.1 µmol^−1^.L.s^−1^) were defined by dividing 1000 µmol^−1^.L.s^−1^ by 100 and 10000, respectively, in order to simulate reactions that cover a large range of velocities. All the k_f_s used are within the range of values observed for the interactions of biomolecules, which typically vary from 0.001 µmol^−1^.L.s^−1^ to 1000 µmol^−1^.L.s^−1^ ^46,47^. For instance, calcium ions interact with many calcium-binding proteins with rates typically in the range of diffusion-limited reactions^48^. The complex calcium/calmodulin activates many targets with rate constants of binding around 1–10 µmol^−1^.L.s^−1^ ^18,49^. In contrast, protein kinase A, a tetrameric enzyme involved in several signaling processes, has rate constants for the binding of its catalytic and regulatory subunits that vary around 0.5 to 0.05 µmol^−1^.L.s^−1^ ^44^. We estimated the k_b_s for the reactions of the model using equation 3:

3 $${K}_{D}=\frac{{k}_{b}}{{k}_{f}}$$

The concentration of M1-M12 was set to 1 µmol.L^−1^ initially (Figs 1–6). In the simulations showed in Figs 7 and 8, we set the initial concentrations of M1-M12 (M4-M6 in Fig. 8) to 10 µmol.L^−1^ and the concentrations of T_1_-T_12_ and T′_1_-T′_12_ to 1 µmol.L^−1^ (T4-T6 and T4′-T6′ in Fig. 8). The interaction of LM1-LM12 with T_1_-T_12_ and T′_1_-T′_12_ were simulated separately.

To obtain the dose-response curves at steady-state (Figs 1 and 7A,B), we performed the simulations until the reactions had reached steady-state. Then, we annotated the final concentrations of the complexes investigated and the concentration of free L ([L]_free_). To trace the dose-responses curves using square pulses of L, we simulated non-conservative signals of [L]_free_. The durations and peak concentrations of the pulses were set by the simulations and were not changed due to interactions with M1-M12, which prevented the competition among them. We defined the durations of the pulses of L setting 10 ms as our lower limit, which corresponds to fast calcium ion signals^28^. We then systematically increased the durations of the pulses until all complexes LM1-LM12 exhibited K_Dapp_s compatible with their control K_D_s. The data used in the dose-responses curves showed in Figs 3 and 6 corresponded to the peak activations of LM1-LM12 obtained as functions of the peak [L]_free_. In Figs 3 and 6, we varied the peak concentrations of the pulses of L from 0 µmol.L^−1^ to ~450 µmol.L^−1^ to achieve saturation of all complexes LM1-LM12. In Fig. 7, we varied the peak concentrations of the pulses of L from 0 µmol.L^−1^ to ~200 µmol.L^−1^ to saturate the complexes LM1T_1_-LM12T_12_ and LM1T′_1_-LM12T′_12_. Nevertheless, we opted to plot the curves of Figs 3, 6 and 7 with a smaller range of concentrations of [L]_free_ for better visualization. We fitted the dose-response curves showed in Figs 3, 7 and 8 and Suppl. Figs S4, S5 and S8 using Matlab curve fitting tool with 95% of confidence interval. The full description of the reactions and the parameters used in the models are listed in Suppl. Table S4.

## Electronic supplementary material

Supplementary Material
