# Publisher Correction: Molecular mechanisms of detection and discrimination of dynamic signals

**DOI:** 10.1038/s41598-018-24192-7

**Published:** 2018-04-17

**Authors:** G. Antunes, A. C. Roque, F. M. Simoes-de-Souza

**Affiliations:** 1 Laboratory of Neural Systems (SisNe), Department of Physics, University of São Paulo, Ribeirão Preto, SP Brazil; 2 Center for Mathematics, Computation and Cognition, Federal University of ABC, São Bernardo do Campo, SP Brazil

Correction to: *Scientific Reports* 10.1038/s41598-018-20842-y, published online 06 February 2018

The original PDF version of this Article contained missing arrows in chemical equations 2 and 3. This has now been corrected in the PDF; the HTML version of the paper was correct from the time of publication.

