# Supplementary material for: Molecular mechanisms of detection and discrimination of dynamic signals
Source: Sci Rep. 2018 Feb 6;8:2480. doi: 10.1038/s41598-018-20842-y (PMC5802782; doi:10.1038/s41598-018-20842-y)
Supplement: Supplementary file 1 — Supplementary Material [file 41598_2018_20842_MOESM1_ESM.pdf]

Title: Title: Molecular mechanisms of detection and discrimination of dynamic signals

Authors: G. Antunes[1], A. C. Roque[1], F. M. Simoes-de-Souza[2]*

Affiliations:

[1]Laboratory of Neural Systems (SisNe), Department of Physics, Faculdade de Filosofia Ciências e Letras de Ribeirão Preto, Universidade de São Paulo, Ribeirão Preto, SP, Brazil

[2]Center for Mathematics, Computation and Cognition, Federal University of ABC, São Bernardo do Campo, SP, Brazil

*To whom correspondence should be addressed: fabio.souza@ufabc.edu.br

Supplementary Figure S1: Time courses of trains of $[L]_{free}$.

Supplementary Figure S2: Examples of the time courses for the formation of LM1-LM12 obtained for trains of pulses of $[L]_{free}$.

Supplementary Figure S3: Schematic representation of how we obtained the data to plot the dose-response curves for trains of pulses of $[L]_{free}$.

Supplementary Figure S4: Dose-response curves for trains of pulses of $[L]_{free}$ with 50 ms of duration.

Supplementary Figure S5: Dose-response curves for trains of pulses of $[L]_{free}$ with 100 ms of duration.

Supplementary Figure S6: n_{Hill} obtained from the curves showed in Supplementary Fig. 4 (A) and 5 (B).

Supplementary Figure S7: K_{Dapp}/K_D ratios calculated using the K_{Dapp}s showed in Fig. 6A (A) and B (B) and the control K_Ds (Fig. 1C).

Supplementary Figure S8: Values of n_{Hill} estimated from the dose-response curves showed in Fig. 7A and B.

Supplementary Table S1: K_{Dapp}s for the activations of LM1-LM12 by pulses of $[L]_{free}$ with different durations estimated from the curves showed in Fig. 3A.

Supplementary Table S2: K_{Dapp}s of the dose-response curves showed in Fig.7A-B.

Supplementary Table S3: Parameters of the dose-response curves showed in Fig.8E-F.

Supplementary Table S4: Reactions and parameters used in the computational models

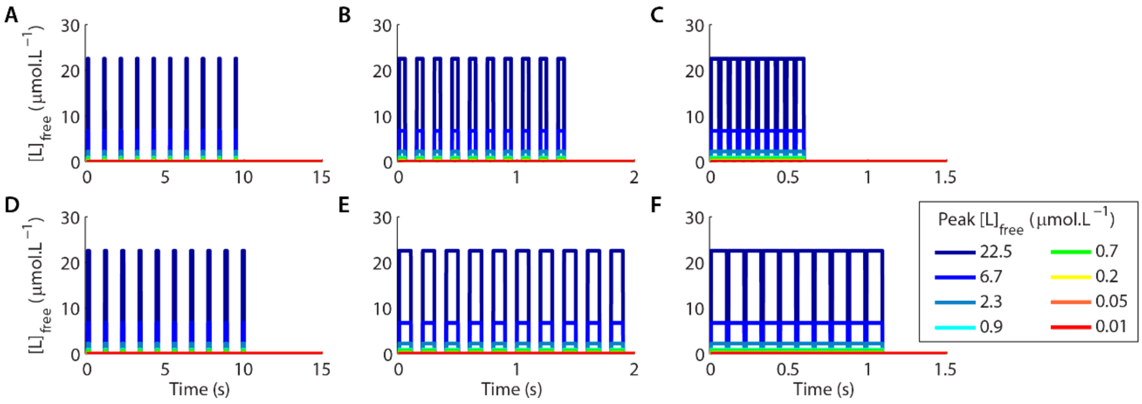

Supplementary Figure S1: Time courses of trains of $[L]_{free}$. The trains consisted of pulses of 50 ms of duration released at 1Hz (**A**), 10 Hz (**B**) and 100 Hz (**C**), or pulses of 100 ms of duration released at the same frequencies (1 Hz: **D**, 10 Hz: **E**, 100 Hz: **F**).

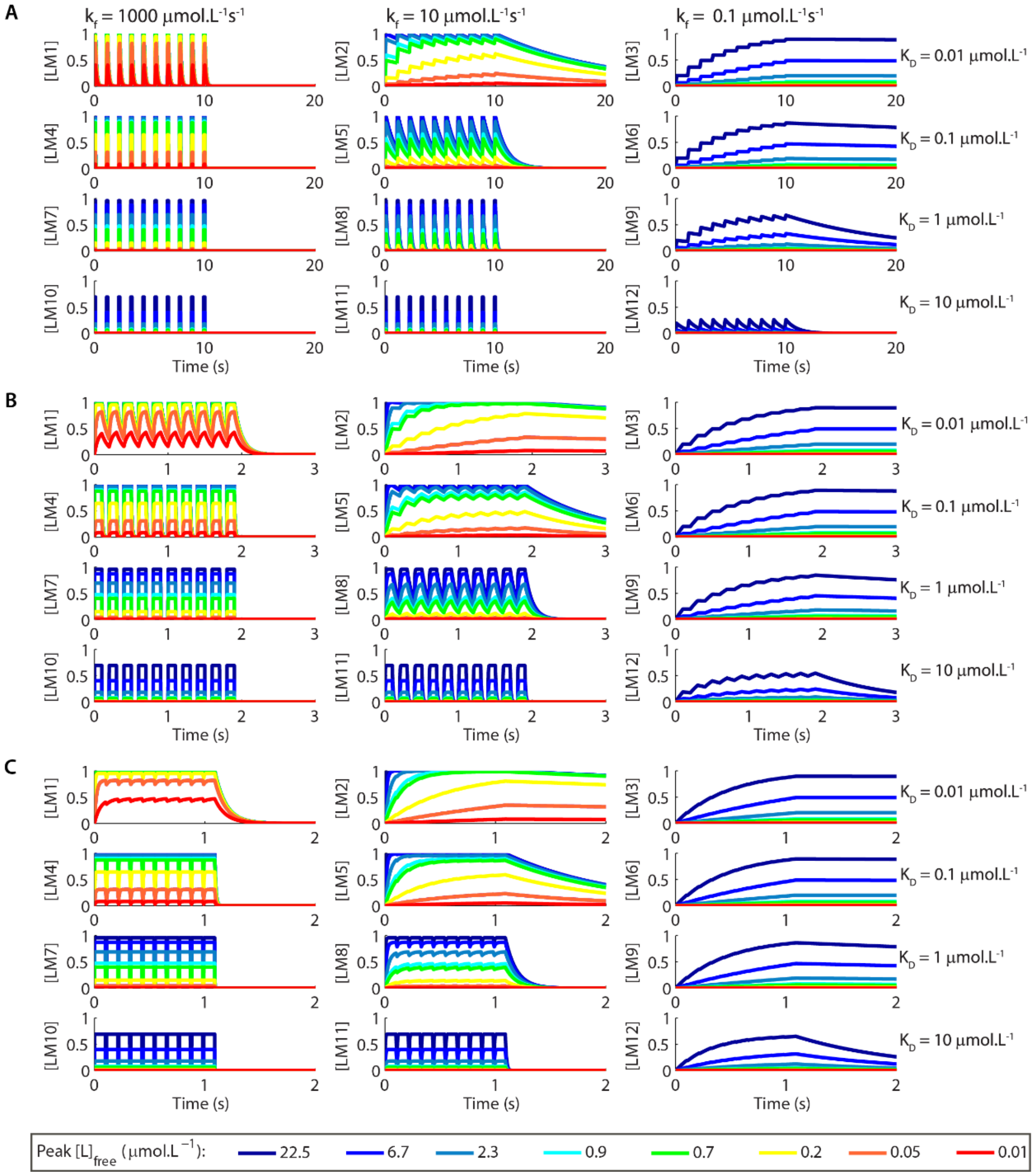

Supplementary Figure S2: Examples of the time courses for the formation of LM1-LM12 obtained for trains of pulses of $[L]_{free}$. The trains consisted of ten pulses (100 ms of duration each) released at 1 Hz **(A)**, 10 Hz **(B)**, or 100 Hz **(C)**. The control K_Ds for the interactions of M1-M12 with L are showed on the right of the panels and the k_fs for the association reactions are indicated in the top of **A**. The k_b for each reaction is calculated by: $k_b = k_f \times K_D$.

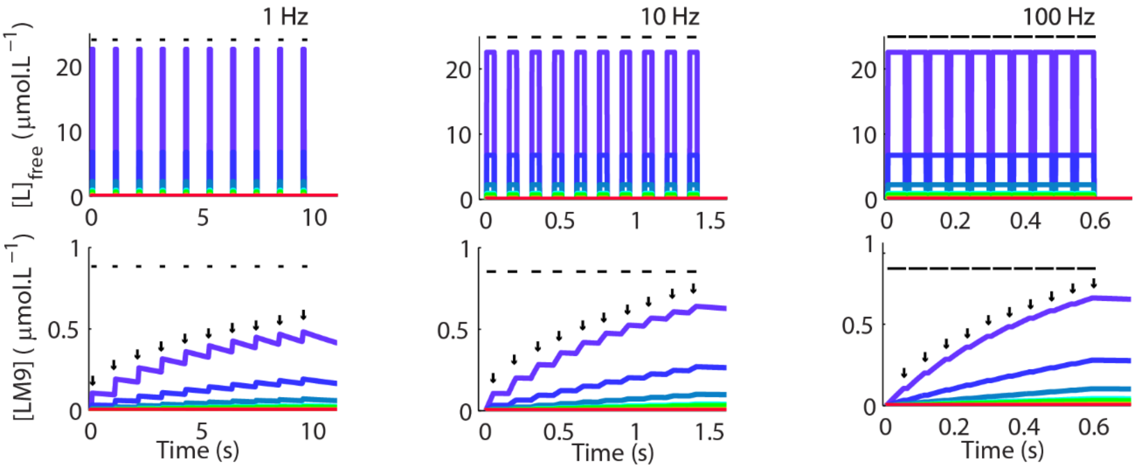

Supplementary Figure S3: Schematic representation of how we obtained the data to plot the dose-response curves for trains of pulses of $[L]_{free}$. To trace the dose-response curves for trains of $[L]_{free}$, for each pulse of $[L]_{free}$ we annotated the peak concentration (indicated by arrows) of LM1-LM12 formed within the duration of the pulse (black lines) and plotted it as a function of the peak $[L]_{free}$. Then, we plotted a dose-response curve for the first pulse of the train, another curve for the second pulse, and so on. With this analysis, we could verify the dynamic changes of K_{Daap} during the trains of signals of L.

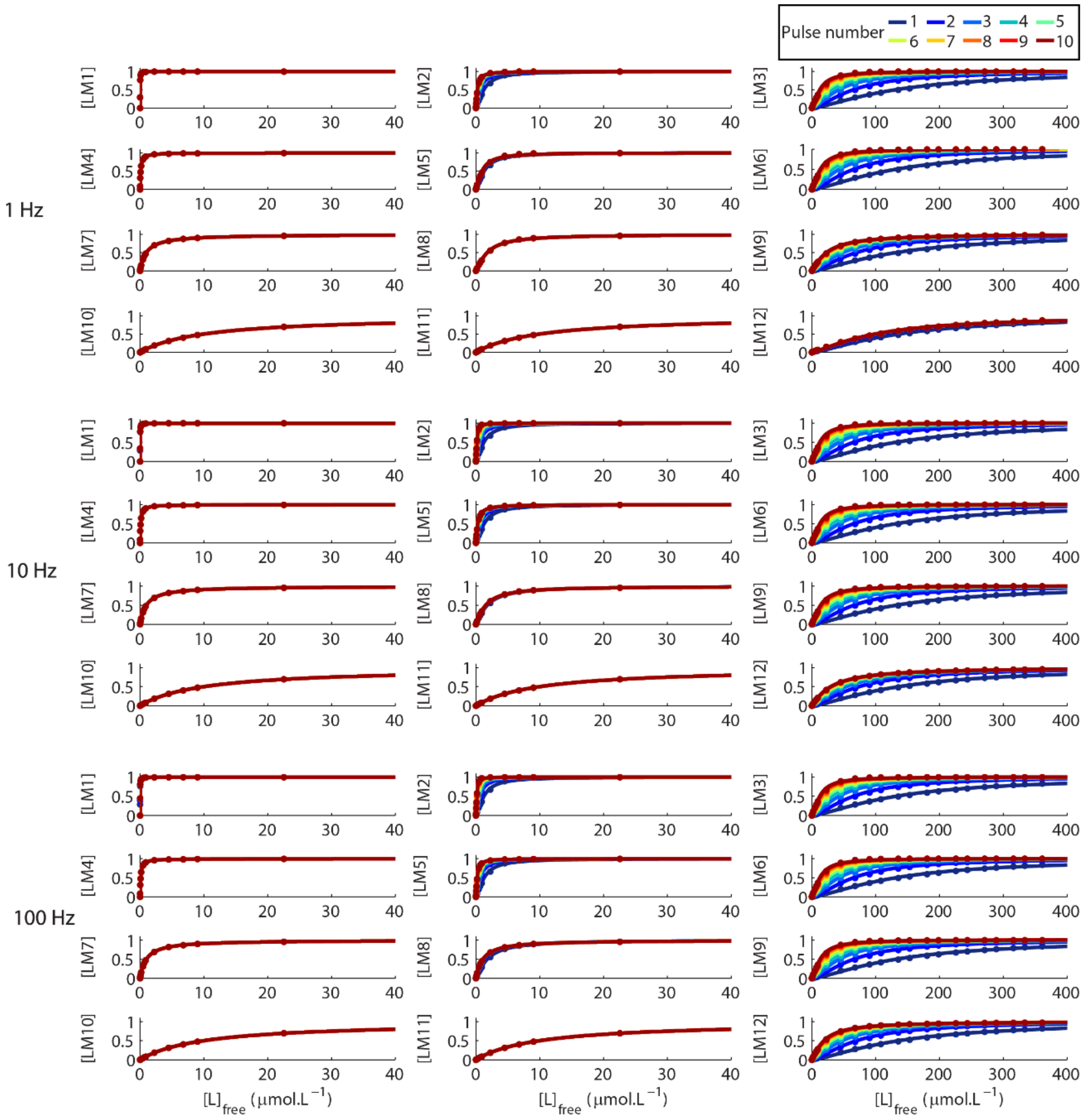

Supplementary Figure S4: Dose-response curves for trains of pulses of $[L]_{free}$ with 50 ms of duration.

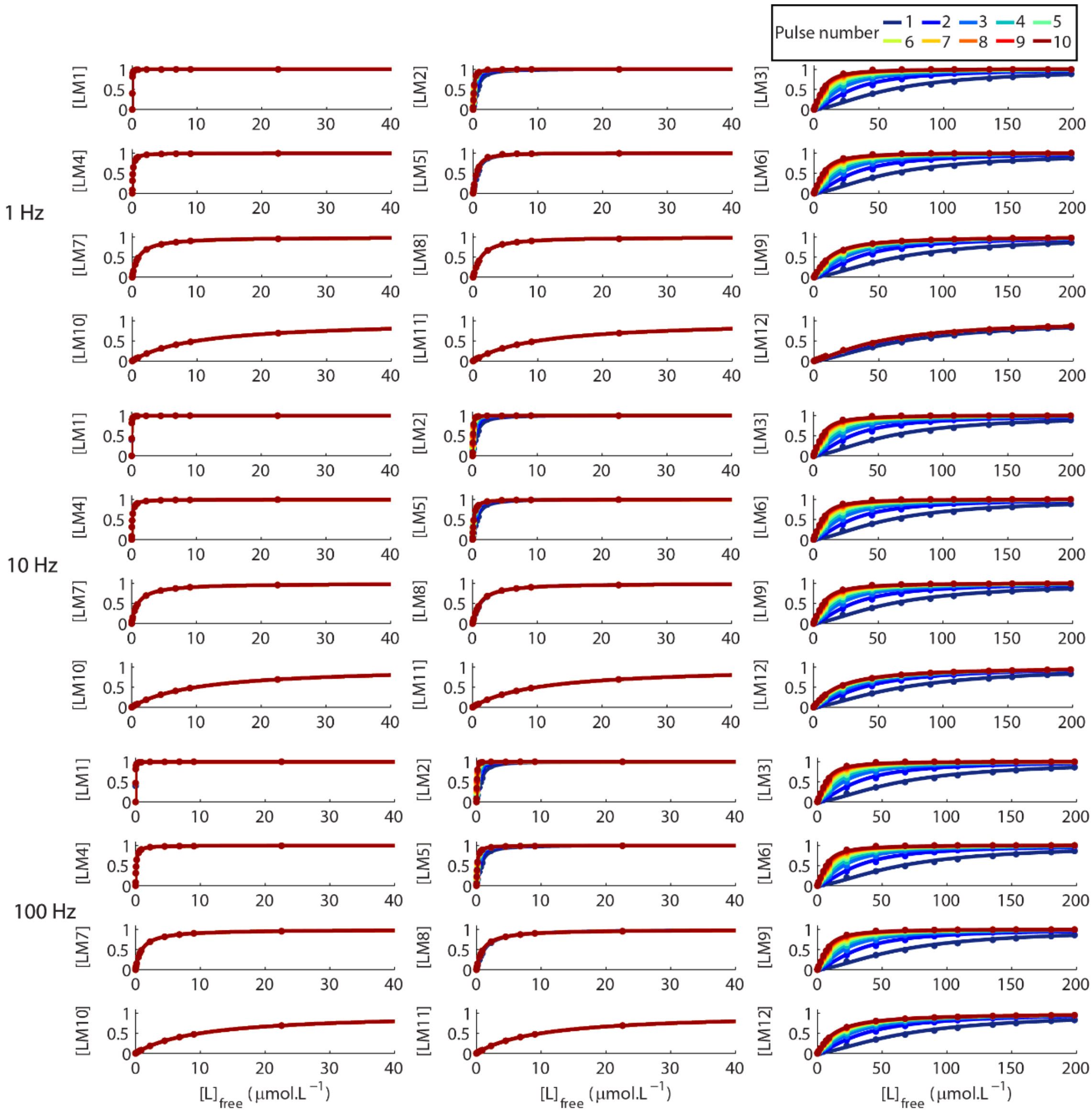

Supplementary Figure S5: Dose-response curves for trains of pulses of $[L]_{free}$ with 100 ms of duration.

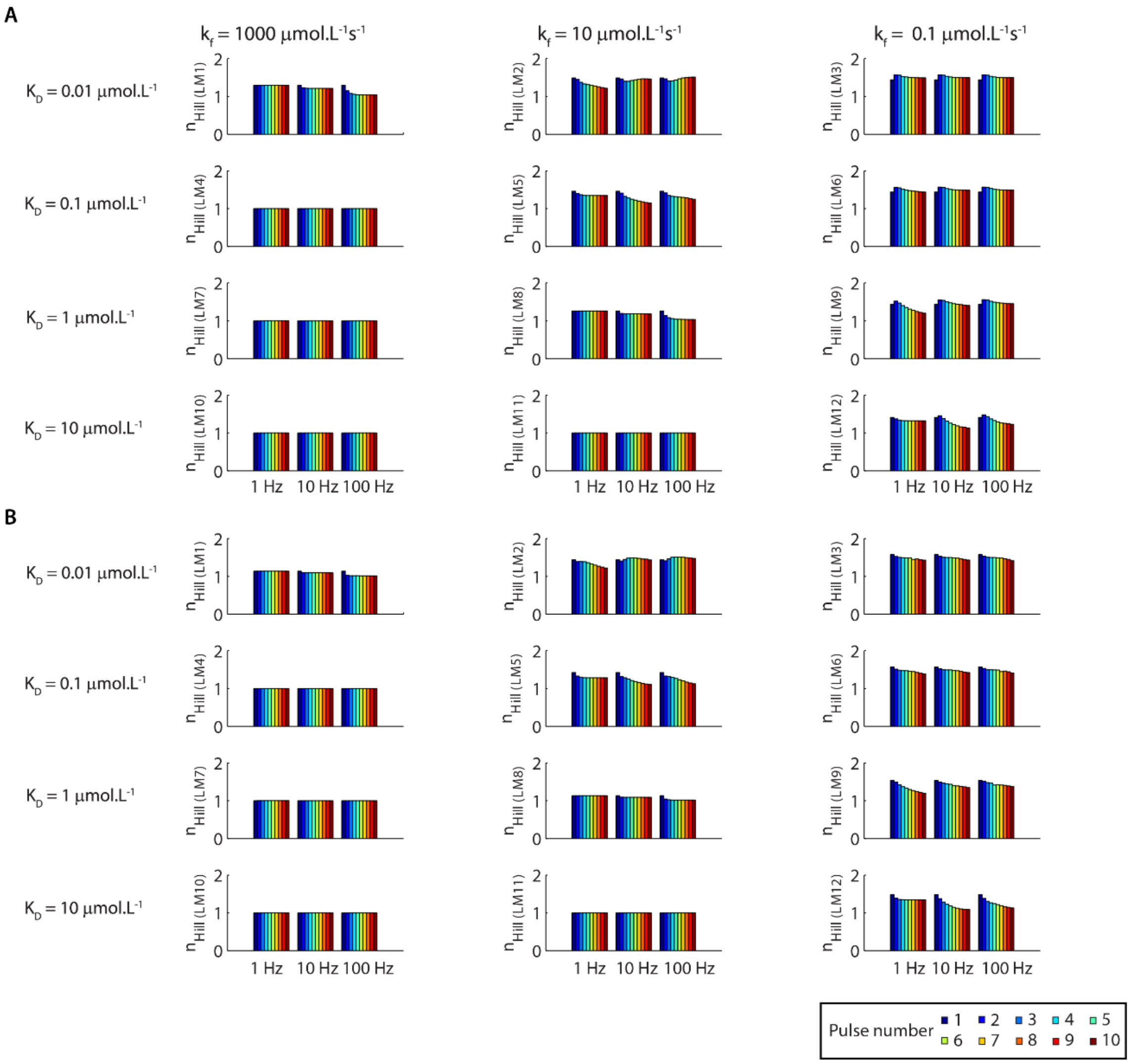

Supplementary Figure S6: n_{Hill} obtained from the curves showed in Supplementary Fig. 4 **(A)** and 5 **(B)**.

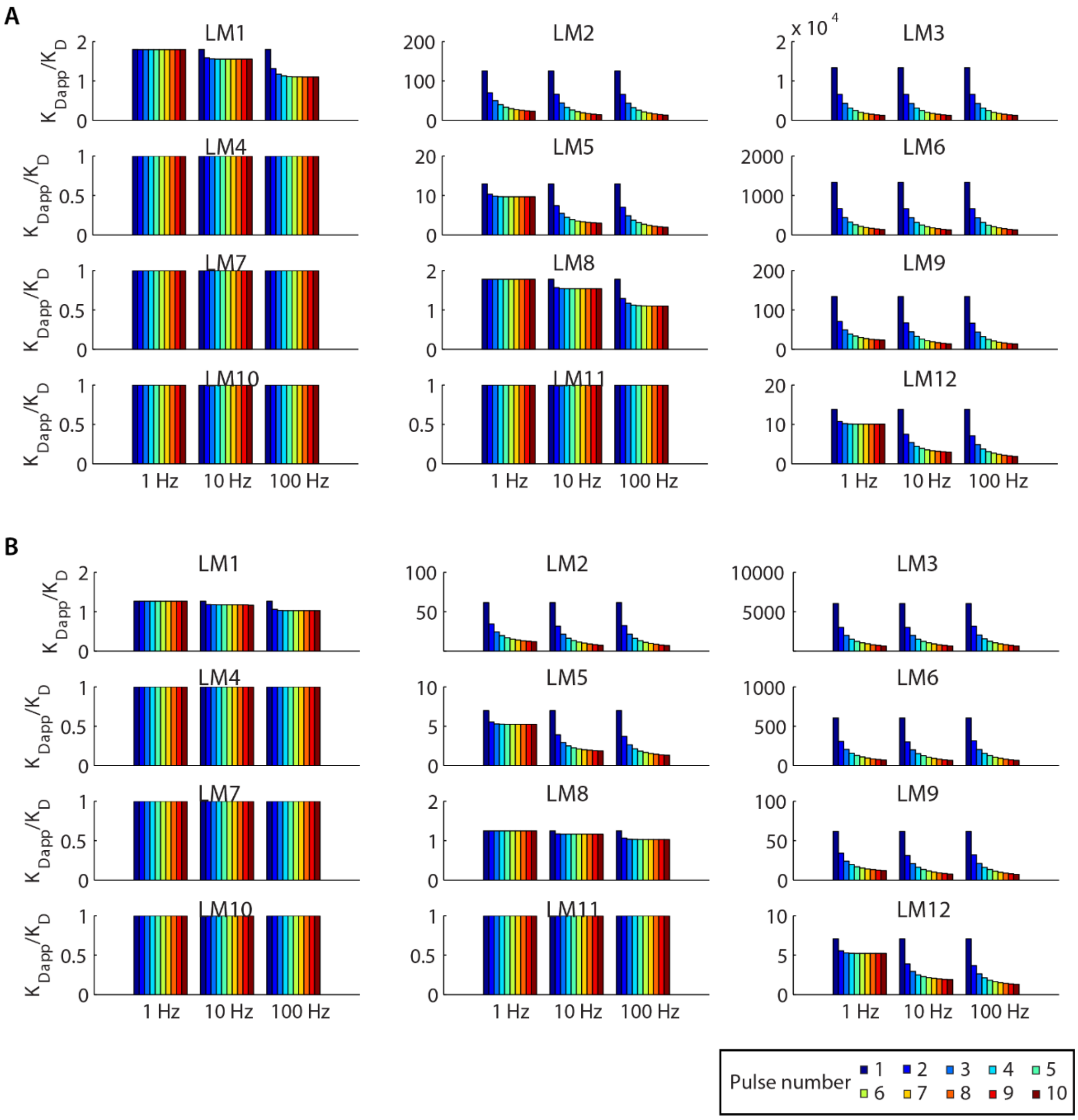

Supplementary Figure S7: K_{Dapp}/K_D ratios calculated using the K_{Dapp}s showed in Fig. 6A **(A)** and B **(B)** and the control K_Ds (Fig. 1C).

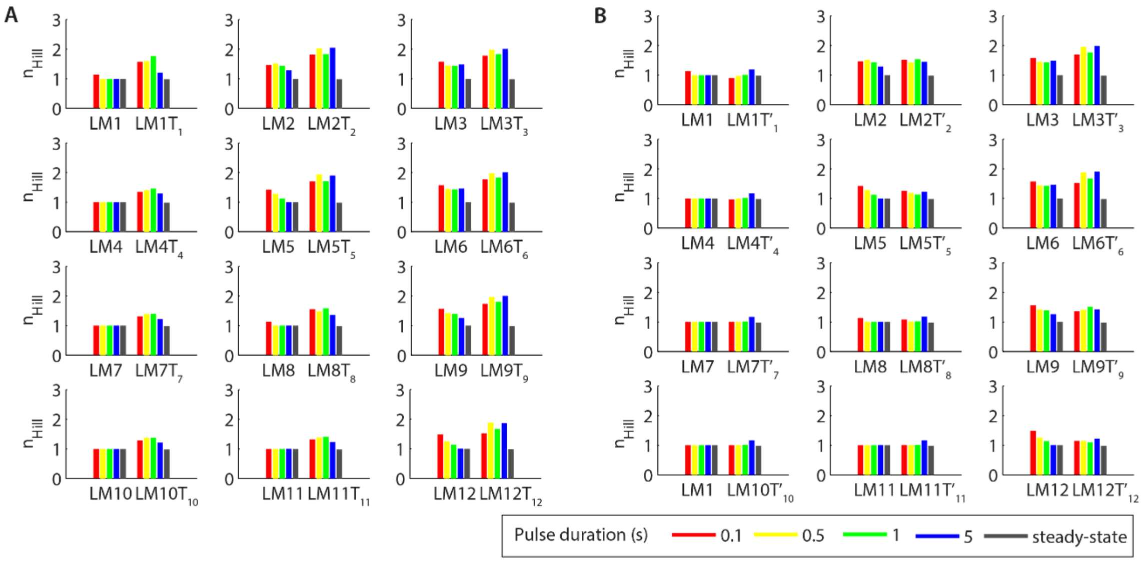

Supplementary Figure S8: Values of n_{Hill} estimated from the dose-response curves showed in Fig. 7A and B. **(A)** Values of n_{Hill} estimated for the formations of $LM1T_1$-$LM12T_{12}$ as functions of pulses of $[L]_{free}$ with different durations. **(B)** n_{Hill} for the formations of the complexes LM1T'$_1$-LM12T'$_{12}$. In **(A)** and **(B)**, we also replotted the values of n_{Hill} for the formation of the binary complexes LM1-LM12 for comparisons. The legend shows the colour code used to represent durations of the pulses of $[L]_{free}$.

Supplementary Table S1: K_{Dapp}s for the activations of LM1-LM12 by pulses of $[L]_{free}$ with different durations estimated from the curves showed in Fig. 3A.

**Pulse duration**	**LM1 K_{Dapp}** (µmol.L^{-1})	**LM2 K_{Dapp}** (µmol.L^{-1})	**LM3 K_{Dapp}** (µmol.L^{-1})
10 ms	0.07084	6.57	660.2
50 ms	0.01797	1.255	133.4
100 ms	0.01266	0.6593	66.02
500 ms	0.01	0.132	12.82
1 s	0.01	0.07043	6.614
5 s	0.01	0.0179	1.254
10 s	0.01	0.0126	0.665
25 s	0.01	0.0101	0.2561
50 s	0.01	0.01	0.132
100 s	0.01	0.01	0.07048
200 s	0.01	0.01	0.03682
300 s	0.01	0.01	0.02698
600 s	0.01	0.01	0.01609
1000 s	0.01	0.01	0.01261
1500 s	0.01	0.01	0.011
**Pulse duration**	**LM4 K_{Dapp}** (µmol.L^{-1})	**LM5 K_{Dapp}** (µmol.L^{-1})	**LM6 K_{Dapp}** (µmol.L^{-1})
10 ms	0.1257	6.616	660.3
50 ms	0.1001	1.295	133.4
100 ms	0.1	0.699	66.06
500 ms	0.1	0.1797	12.86
1 s	0.1	0.1257	6.652
5 s	0.1	0.1001	1.295
10 s	0.1	0.1	0.7036
25 s	0.1	0.1	0.3025
50 s	0.1	0.1	0.1795
100 s	0.1	0.1	0.1248
200 s	0.1	0.1	0.1041
300 s	0.1	0.1	0.1
600 s	0.1	0.1	0.1
1000 s	0.1	0.1	0.1
1500 s	0.1	0.1	0.1
**Pulse duration**	**LM7 K_{Dapp}** (µmol.L^{-1})	**LM8 K_{Dapp}** (µmol.L^{-1})	**LM9 K_{Dapp}** (µmol.L^{-1})
10 ms	1	7.01	660.8
50 ms	1	1.778	133.9
100 ms	1	1.246	66.47
500 ms	1	1.001	13.23
1 s	1	1	7.035
5 s	1	1	1.777
10 s	1	1	1.242
25 s	1	1	1.018
50 s	1	1	1

100 s	1	1	1
200 s	1	1	1
300 s	1	1	1
600 s	1	1	1
1000 s	1	1	1
1500 s	1	1	1
**Pulse duration**	**LM10 K_{Dapp}** (µmol.L $^{-1}$)	**LM11 K_{Dapp}** (µmol.L $^{-1}$)	**LM12 K_{Dapp}** (µmol.L $^{-1}$)
10 ms	10	12.4	665.9
50 ms	10	10	138.2
100 ms	10	10	70.63
500 ms	10	10	17.72
1 s	10	10	12.31
5 s	10	10	10
10 s	10	10	10
25 s	10	10	10
50 s	10	10	10
100 s	10	10	10
200 s	10	10	10
300 s	10	10	10
600 s	10	10	10
1000 s	10	10	10
1500 s	10	10	10

Supplementary Table S2: K_{Dapp}s of the dose-response curves showed in Fig.7A-B.

**Pulse duration**	**LM1T$_1$ K_{Dapp}** (µmol.L^{-1})	**LM2T$_2$ K_{Dapp}** (µmol.L^{-1})	**LM3T$_3$ K_{Dapp}** (µmol.L^{-1})
100 ms	0.001131	0.04952	4.847
500 ms	0.0002414	0.009404	0.9416
1 s	0.000122	0.004949	0.4861
5 s	2.006e-05	0.0009403	0.09309
Steady-state (K_D)	1.052e-05	1.052e-05	1.052e-05
**Pulse duration**	**LM4T$_4$ K_{Dapp}** (µmol.L^{-1})	**LM5T$_5$ K_{Dapp}** (µmol.L^{-1})	**LM6T$_6$ K_{Dapp}** (µmol.L^{-1})
100 ms	0.007818	0.05742	4.857
500 ms	0.001458	0.01044	0.9429
1 s	0.0007806	0.005735	0.4872
5 s	0.0001996	0.00107	0.09324
Steady-state (K_D)	0.0001053	0.0001053	0.0001053
**Pulse duration**	**LM7T$_7$ K_{Dapp}** (µmol.L^{-1})	**LM8T$_8$ K_{Dapp}** (µmol.L^{-1})	**LM9T$_9$ K_{Dapp}** (µmol.L^{-1})
100 ms	0.07537	0.1161	4.948
500 ms	0.0137	0.02302	0.9529
1 s	0.007333	0.01154	0.4965
5 s	0.001859	0.00273	0.09445
Steady-state (K_D)	0.001054	0.00105	0.001054
**Pulse duration**	**LM10T$_{10}$ K_{Dapp}** (µmol.L^{-1})	**LM11T$_{11}$ K_{Dapp}** (µmol.L^{-1})	**LM12T$_{12}$ K_{Dapp}** (µmol.L^{-1})
100 ms	0.7506	0.7932	5.805
500 ms	0.1359	0.1449	1.059
1 s	0.0729	0.0774	0.578
5 s	0.01845	0.01927	0.1073
Steady-state (K_D)	0.01053	0.01053	0.01053
**Pulse duration**	**LM1T$_1$ K_{Dapp}** (µmol.L^{-1})	**LM2T$_2$ K_{Dapp}** (µmol.L^{-1})	**LM3T$_3$ K_{Dapp}** (µmol.L^{-1})
100 ms	0.02022	0.1172	4.981
500 ms	0.009255	0.02354	0.9562
1 s	0.006467	0.01165	0.4999
5 s	0.001564	0.002329	0.09463
Steady-state	1.052e-05	1.052e-05	1.052e-05
**Pulse duration**	**LM4T'$_4$ K_{Dapp}** (µmol.L^{-1})	**LM5T'$_5$ K_{Dapp}** (µmol.L^{-1})	**LM6T'$_6$ K_{Dapp}** (µmol.L^{-1})
100 ms	0.1094	0.5289	5.808
500 ms	0.07848	0.1286	1.06
1 s	0.06071	0.07621	0.5776
5 s	0.01562	0.01613	0.1047
Steady-state	0.0001053	0.0001053	0.0001053
**Pulse duration**	**LM7T'$_7$ K_{Dapp}** (µmol.L^{-1})	**LM8T'$_8$ K_{Dapp}** (µmol.L^{-1})	**LM9T'$_9$ K_{Dapp}** (µmol.L^{-1})
100 ms	0.9596	1.545	12.66
500 ms	0.7702	0.8697	2.375
1 s	0.6034	0.6345	1.186
5 s	0.1568	0.1574	0.2354

Steady-state	0.001054	0.001054	0.001054
**Pulse duration**	**LM10T'$_{10}$ K$_{Dapp}$** (µmol.L $^{-1}$)	**LM11T'$_{s11}$ K$_{Dapp}$** (µmol.L $^{-1}$)	**LM12T'$_{12}$ K$_{Dapp}$** (µmol.L $^{-1}$)
100 ms	9.49	10.33	51.8
500 ms	7.689	7.804	13.27
1 s	6.033	6.068	7.803
5 s	1.569	1.569	1.623
Steady-state	0.01053	0.01053	0.01053

Supplementary Table S3: Parameters of the dose-response curves showed in Fig.8E-F

**Pulse duration**	**LM4T$_4$**	**LM4T'$_4$**
0.5 s	A_{max} = 1.0*<br>K_{Dapp} = 0.0014<br>n_{Hill} = 1.4912<br><br>without competition:<br>A_{max} =1.0<br>K_{Dapp} = 0.00148<br>n_{Hill} = 1.493	A_{max} = 0.36<br>K_{Dapp} = 0.079<br>n_{Hill} = 1<br><br>without competition:<br>A_{max} = 0.39<br>K_{Dapp} = 0.078<br>n_{Hill} = 1
5.0 s	A_{max} = 0.99<br>K_{Dapp} = 0.00017<br>n_{Hill} = 1.119<br><br>without competition:<br>A_{max} = 0.998<br>K_{Dapp} = 0.00019<br>n_{Hill} = 1.29	A_{max} = 0.98<br>K_{Dapp} = 0.017<br>n_{Hill} = 1.17<br><br>without competition:<br>A_{max} = 0.99<br>K_{Dapp} = 0.015<br>n_{Hill} = 1.19
**Pulse duration**	**LM5T$_5$**	**LM5T'$_5$**
0.5 s	A_{max} = 1.0<br>K_{Dapp} = 0.011<br>n_{Hill} = 1.95<br><br>without competition:<br>A_{max} = 1.0<br>K_{Dapp} = 0.0109<br>n_{Hill} = 1.94	A_{max} = 0.71<br>K_{Dapp} = 0.14<br>n_{Hill} = 1.22<br><br>without competition:<br>A_{max} = 0.75<br>K_{Dapp} = 0.12<br>n_{Hill} = 1.19
5.0 s	A_{max} = 0.99<br>K_{Dapp} = 0.00093<br>n_{Hill} =2.09<br><br>without competition:<br>A_{max} = 1.0	A_{max} = 0.99<br>K_{Dapp} = 0.019<br>n_{Hill} = 1.26<br><br>without competition:<br>A_{max} = 0.99

	K_{Dapp} = 0.0011<br>n_{Hill} = 1.84	K_{Dapp} = 0.016<br>n_{Hill} = 1.25
**Pulse duration**	**LM6T$_6$**	**LM6T'$_6$**
0.5 s	A_{max} = 1.02<br>K_{Dapp} = 0.98<br>n_{Hill} = 1.88<br><br>without competition:<br>A_{max} = 1.02<br>K_{Dapp} = 0.97<br>n_{Hill} = 1.96	A_{max} = 1.0<br>K_{Dapp} = 2.09<br>n_{Hill} = 1.73<br><br>without competition:<br>A_{max} = 1.0<br>K_{Dapp} = 1.09<br>n_{Hill} = 1.83
5.0 s	A_{max} = 1.0<br>K_{Dapp} = 0.096<br>n_{Hill} = 1.96<br><br>without competition:<br>A_{max} = 1.0<br>K_{Dapp} = 0.094<br>n_{Hill} = 1.99	A_{max} = 1.0<br>K_{Dapp} = 0.20<br>n_{Hill} = 1.84<br><br>without competition:<br>A_{max} = 1.0<br>K_{Dapp} = 0.10<br>n_{Hill} = 1.93

* A_{max} is given in µmol.L^{-1}

Supplementary Table S4: Reactions and parameters used in the models of ternary complexes formation/dissociation

**Interactions of M1-M12 with T_{f1}-T_{f12}**	**Interactions of M1-M12 with T_{s1}-T_{s12}**
$LM1 + T_{f1} \underset{0.1 s^{-1}}{\overset{10 \mu mol^{-1}.L.s^{-1}}{\rightleftharpoons}} LM1T_1$	$LM1 + T'_1 \underset{0.001 s^{-1}}{\overset{0.1 \mu mol^{-1}.L.s^{-1}}{\rightleftharpoons}} LM1T'_1$
$LM2 + T_2 \underset{0.1 s^{-1}}{\overset{10 \mu mol^{-1}.L.s^{-1}}{\rightleftharpoons}} LM2T_2$	$LM2 + T'_2 \underset{0.001 s^{-1}}{\overset{0.1 \mu mol^{-1}.L.s^{-1}}{\rightleftharpoons}} LM2T'_2$
$LM3 + T_3 \underset{0.1 s^{-1}}{\overset{10 \mu mol^{-1}.L.s^{-1}}{\rightleftharpoons}} LM3T_3$	$LM3 + T'_3 \underset{0.001 s^{-1}}{\overset{0.1 \mu mol^{-1}.L.s^{-1}}{\rightleftharpoons}} LM3T'_3$
$LM4 + T_4 \underset{0.1 s^{-1}}{\overset{10 \mu mol^{-1}.L.s^{-1}}{\rightleftharpoons}} LM4T_4$	$LM4 + T'_4 \underset{0.001 s^{-1}}{\overset{0.1 \mu mol^{-1}.L.s^{-1}}{\rightleftharpoons}} LM4T'_4$
$LM5 + T_5 \underset{0.1 s^{-1}}{\overset{10 \mu mol^{-1}.L.s^{-1}}{\rightleftharpoons}} LM5T_5$	$LM5 + T'_5 \underset{0.001 s^{-1}}{\overset{0.1 \mu mol^{-1}.L.s^{-1}}{\rightleftharpoons}} LM5T'_5$
$LM6 + T_6 \underset{0.1 s^{-1}}{\overset{10 \mu mol^{-1}.L.s^{-1}}{\rightleftharpoons}} LM6T_6$	$LM6 + T'_6 \underset{0.001 s^{-1}}{\overset{0.1 \mu mol^{-1}.L.s^{-1}}{\rightleftharpoons}} LM6T'_6$
$LM7 + T_7 \underset{0.1 s^{-1}}{\overset{10 \mu mol^{-1}.L.s^{-1}}{\rightleftharpoons}} LM7T_7$	$LM7 + T'_7 \underset{0.001 s^{-1}}{\overset{0.1 \mu mol^{-1}.L.s^{-1}}{\rightleftharpoons}} LM7T'_7$
$LM8 + T_8 \underset{0.1 s^{-1}}{\overset{10 \mu mol^{-1}.L.s^{-1}}{\rightleftharpoons}} LM8T_8$	$LM8 + T'_8 \underset{0.001 s^{-1}}{\overset{0.1 \mu mol^{-1}.L.s^{-1}}{\rightleftharpoons}} LM8T'_8$
$LM9 + T_9 \underset{0.1 s^{-1}}{\overset{10 \mu mol^{-1}.L.s^{-1}}{\rightleftharpoons}} LM9T_9$	$LM9 + T'_9 \underset{0.001 s^{-1}}{\overset{0.1 \mu mol^{-1}.L.s^{-1}}{\rightleftharpoons}} LM9T'_9$
$LM10 + T_{10} \underset{0.1 s^{-1}}{\overset{10 \mu mol^{-1}.L.s^{-1}}{\rightleftharpoons}} LM10T_{10}$	$LM10 + T'_{10} \underset{0.001 s^{-1}}{\overset{0.1 \mu mol^{-1}.L.s^{-1}}{\rightleftharpoons}} LM10T'_{10}$
$LM11 + T_{11} \underset{0.1 s^{-1}}{\overset{10 \mu mol^{-1}.L.s^{-1}}{\rightleftharpoons}} LM11T_{11}$	$LM11 + T'_{11} \underset{0.001 s^{-1}}{\overset{0.1 \mu mol^{-1}.L.s^{-1}}{\rightleftharpoons}} LM11T'_{11}$
$LM12 + T_{12} \underset{0.1 s^{-1}}{\overset{10 \mu mol^{-1}.L.s^{-1}}{\rightleftharpoons}} LM12T_{12}$	$LM12 + T_{12} \underset{0.001 s^{-1}}{\overset{0.1 \mu mol^{-1}.L.s^{-1}}{\rightleftharpoons}} LM12T_{12}$
